# METTL3‐Mediated N6‐Methyladenosine Modification Activates the EGR1‐ZBP1 Axis to Promote PANoptosis‐Like Cardiomyocyte Death in Myocardial Ischemia‐Reperfusion Injury

**DOI:** 10.1002/mco2.70962

**Published:** 2026-09-21

**Authors:** Linnan Li, Jinfeng Gao, Xiaoxue Zhang, Kunming Dai, Hao Cheng, Jianying Ma, Junbo Ge

**Affiliations:** ^1^ Department of Cardiology Zhongshan Hospital Fudan University Shanghai Institute of Cardiovascular Diseases Shanghai China; ^2^ State Key Laboratory of Cardiovascular Diseases Zhongshan Hospital Fudan University Shanghai China; ^3^ NHC Key Laboratory of Ischemic Heart Diseases Shanghai China; ^4^ Key Laboratory of Viral Heart Diseases Chinese Academy of Medical Sciences Shanghai China; ^5^ National Clinical Research Center for Interventional Medicine Shanghai China; ^6^ Institutes of Biomedical Sciences Fudan University Shanghai China

**Keywords:** methyltransferase‐like 3, myocardial ischemia‐reperfusion injury, N6‐methyladenosine, PANoptosis

## Abstract

Myocardial ischemia‐reperfusion (I/R) injury is a major cause of cardiac dysfunction, but the mechanisms linking metabolic stress to regulated cardiomyocyte death remain incompletely defined. N6‐methyladenosine (m^6^A), the most abundant internal modification of eukaryotic mRNA, is installed by the methyltransferase‐like 3 (METTL3)‐containing writer complex and regulates RNA fate. Here, we investigated whether METTL3‐mediated m^6^A modification contributes to PANoptosis‐like cardiomyocyte death during I/R injury. In mouse myocardial I/R and oxygen‐glucose deprivation/reoxygenation (OGD/R) models, global m^6^A levels and METTL3 expression were markedly increased. Lactate accumulation was associated with p300‐dependent H3K18 lactylation (H3K18la) enrichment at the *Mettl3* promoter, suggesting a metabolic‐epigenetic mechanism for METTL3 transcriptional upregulation. Functionally, METTL3 promoted PANoptosis‐related death signaling, strengthened the association of ZBP1 with PANoptosis‐related components, and aggravated infarction, adverse remodeling, and cardiac dysfunction, whereas METTL3 deletion or pharmacological inhibition with STM2457 was protective. Mechanistically, METTL3 enhanced m^6^A modification of *Egr1* mRNA, thereby increasing EGR1 expression through YTHDF1‐mediated translational regulation and IGF2BP2‐mediated mRNA stabilization. EGR1 further activated *Zbp1* transcription, and EGR1 restoration partially reversed the protective effects of METTL3 deficiency. These findings identify a lactate‐H3K18la‐METTL3‐EGR1‐ZBP1 axis that drives PANoptosis‐like cardiomyocyte death and suggest METTL3 inhibition as a potential strategy against I/R‐induced cardiac injury.

## Graphical abstract

Myocardial ischemia‐reperfusion injury promotes lactate accumulation and H3K18 lactylation at the *Mettl3* promoter, inducing METTL3 expression in cardiomyocytes. METTL3 installs m^6^A on *Egr1* mRNA, enhancing EGR1 expression via YTHDF1‐mediated translation and IGF2BP2‐dependent stabilization. EGR1 activates *Zbp1* transcription, facilitating ZBP1‐associated PANoptosome assembly and PANoptosis‐like cardiomyocyte death. STM2457 inhibits METTL3 and attenuates this axis to protect against cardiac injury.

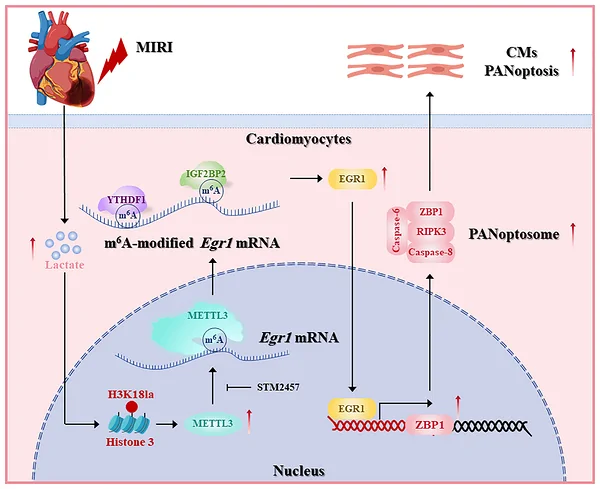

## 1 Introduction

Acute myocardial infarction (AMI) remains a major global health burden. Although rapid reperfusion is the most effective strategy for limiting ischemic injury and infarct size, restoration of blood flow can itself cause additional and often irreversible myocardial damage [1, 2, 3, 4]. Myocardial ischemia‐reperfusion (I/R) injury involves oxidative stress, inflammation, calcium overload, endothelial dysfunction, and microvascular impairment [5]. Despite advances in mechanical and pharmacological interventions, no effective therapy specifically targeting reperfusion injury has been established [6, 7, 8], highlighting the need for new regulatory mechanisms and therapeutic targets.

Epigenetics refers to the regulation of gene expression without alterations in the DNA sequence and involves reversible and dynamic modifications at the DNA, histone, and RNA levels [9, 10]. These modifications primarily include DNA methylation, histone acetylation, RNA modifications, and chromatin remodeling, all of which affect various biological processes such as gene expression, protein function, aging, and disease pathogenesis [11]. N6‐methyladenosine (m^6^A), the most prevalent internal modification in eukaryotic mRNA [12, 13], regulates RNA splicing, nuclear export, stability, translation, and degradation [14]. It is dynamically regulated by “writers,” “readers,” and “erasers” [15, 16]. METTL3, the catalytic core of the m^6^A methyltransferase complex, is the most extensively studied writer [17].

Accumulating evidence implicates m^6^A modification in cardiovascular diseases, including cardiac hypertrophy, hypertension, and heart failure [18, 19, 20]. In myocardial I/R injury, METTL3‐mediated m^6^A modification of *Tfeb* suppresses autophagy and promotes cardiomyocyte apoptosis [21], whereas WTAP enhances *Atf4* m^6^A modification, endoplasmic reticulum stress, and apoptosis in OGD/R‐treated cardiomyocytes [22]. However, the upstream signals driving m^6^A remodeling and the critical downstream effector networks remain poorly defined.

PANoptosis is an inflammatory form of programmed cell death coordinated by innate immune sensors, caspases, and receptor‐interacting protein kinases [23, 24]. It integrates molecular features of pyroptosis, apoptosis, and necroptosis [25, 26] and is associated with the assembly of a multiprotein PANoptosome [27]. Four major PANoptosome models have been described, containing ZBP1, AIM2, RIPK1, or NLRP1 [28, 29]. Among them, the ZBP1‐PANoptosome is the most extensively characterized and was first identified during influenza A virus infection [29, 30].

Apoptosis, pyroptosis, and necroptosis all contribute to cardiomyocyte death during I/R injury [31, 32]. Inhibiting a single pathway may provide insufficient cardioprotection or permit compensatory activation of others [33], suggesting that these death programs are interconnected through shared upstream signals and regulatory nodes. PANoptosis may therefore offer a more integrated framework for understanding and targeting I/R‐induced cardiomyocyte death.

To date, most studies on PANoptosis have focused on cancer, infectious diseases, and inflammatory disorders [25], consistent with its important role in innate immunity and the association of established PANoptosome models with pathogen infection, inflammation, or cellular stress [34, 35]. Emerging evidence has also implicated PANoptosis‐related mechanisms in several cardiovascular diseases, including doxorubicin‐induced cardiomyopathy, SARS‐CoV‐2‐associated myocarditis, and dilated cardiomyopathy [36, 37, 38]. However, whether PANoptosis‐related death signaling is activated in cardiomyocytes during myocardial I/R injury and how this process is regulated remain unclear.

In this study, we investigated PANoptosis‐related death signaling and PANoptosis‐like cardiomyocyte death during myocardial I/R injury and explored the regulatory role of METTL3‐mediated m^6^A modification. Our findings identify METTL3‐dependent m^6^A signaling as an epitranscriptomic regulator of cardiomyocyte death and a potential therapeutic target in myocardial I/R injury.

## 2 Results

### 2.1 I/R Injury Induces Global m^6^A Accumulation and METTL3 Upregulation In Vivo and In Vitro

**FIGURE 1:**
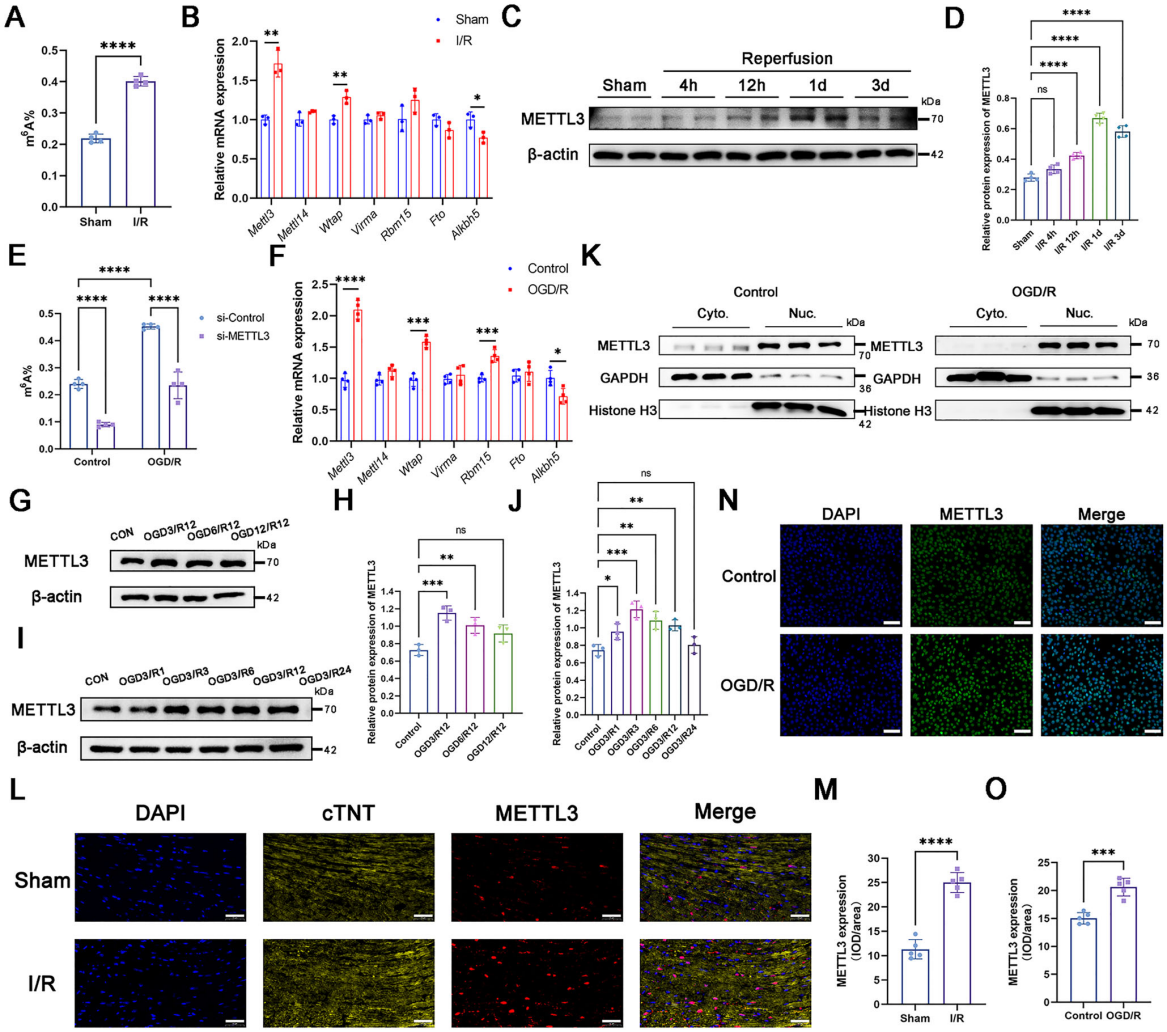
I/R injury induces global m^6^A accumulation and METTL3 upregulation in vivo and in vitro. (A) Relative m^6^A levels in total RNA extracted from myocardial tissue of sham‐operated and I/R‐injured mice at 24 h after reperfusion (*n* = 4). (B) RT‐qPCR analysis of *Mettl3*, *Mettl14*, *Wtap*, *Virma*, *Rbm15*, *Fto*, and *Alkbh5* mRNA levels in myocardial tissue of sham‐operated and I/R‐injured mice at 24 h after reperfusion (*n* = 3). (C, D) Western blot analysis of METTL3 protein levels in myocardial tissue from sham‐operated mice or mice subjected to 45 min of myocardial ischemia followed by 4 h, 12 h, 1 day, or 3 days of reperfusion (*n* = 4). (E) Relative m^6^A levels in total RNA extracted from NMCMs subjected to OGD/R (*n* = 4). (F) RT‐qPCR analysis of *Mettl3*, *Mettl14*, *Wtap*, *Virma*, *Rbm15*, *Fto*, and *Alkbh5* mRNA levels in NMCMs subjected to OGD/R (*n* = 4). (G, H) Western blot analysis of METTL3 protein levels in NMCMs exposed to OGD for 0, 3, 6, or 12 h, followed by 12 h of reoxygenation (*n* = 3). (I, J) Western blot analysis of METTL3 protein levels in NMCMs subjected to OGD for 3 h, followed by 1, 3, 6, 12, or 24 h of reoxygenation (*n* = 3). (K) Western blot analysis of cytoplasmic and nuclear METTL3 protein levels in NMCMs under control or OGD/R conditions (*n* = 3). (L, M) Representative immunofluorescence images depicting METTL3 subcellular distribution in myocardial tissue from sham‐ or I/R‐treated mice (*n* = 5). Scale bar: 50 µm. (N, O) Representative immunofluorescence images showing the subcellular localization of METTL3 in HL‐1 cells under control or OGD/R conditions (*n* = 5). Scale bar: 100 µm. All data are presented as mean ± SD. ns indicates not significant; *p < 0.05; **p < 0.01; ***p < 0.001; ****p < 0.0001.

To investigate the involvement of m^6^A modification in myocardial I/R injury, we quantified global m^6^A levels in myocardial tissue harvested 24 h after reperfusion and found a significant increase (Figure 1A). RT‐qPCR profiling of key m^6^A regulators showed that *Mettl3* and *Wtap* were upregulated, whereas *Alkbh5* was downregulated in I/R‐injured hearts (Figure 1B). Among these regulators, *Mettl3* exhibited the most prominent change. METTL3 protein expression was also increased at multiple reperfusion time points, with the most prominent elevation at 24 h after reperfusion (Figure 1C,D), suggesting that METTL3 may be involved in the early phase of myocardial I/R injury.

Consistent with the in vivo findings, OGD/R increased global m^6^A levels in neonatal mouse cardiomyocytes (NMCMs) (Figure 1E). *Mettl3*, *Wtap*, and *Rbm15* mRNA levels were upregulated, whereas *Alkbh5* mRNA was downregulated (Figure 1F). Among these regulators, *Mettl3* showed the most pronounced change. METTL3 protein levels increased in NMCMs and HL‐1 cells with increasing OGD duration and peaked after 3 h of OGD (Figure 1G,H; Figure S1A,B). With OGD duration fixed, METTL3 increased rapidly after reoxygenation, peaked at 3 h, and remained elevated at several early time points (Figure 1I,J; Figure S1C,D). These temporal changes were consistent with those observed during early reperfusion in vivo. Given its central role as the catalytic component of the m^6^A methyltransferase complex, METTL3 was selected for further investigation.

Nuclear‐cytoplasmic fractionation and immunofluorescence showed that the increased METTL3 expression in I/R‐injured myocardium and OGD/R‐treated cells was predominantly nuclear (Figure 1K–O), consistent with its canonical role as an m^6^A “writer.” Thus, I/R and OGD/R induce global m^6^A accumulation accompanied by robust METTL3 upregulation and predominant nuclear localization, supporting a potential role for METTL3 in the early phase of myocardial I/R injury.

### 2.2 Lactate‐Associated p300‐Dependent H3K18la Contributes to METTL3 Transcriptional Upregulation Following OGD/R and I/R Injury

**FIGURE 2:**
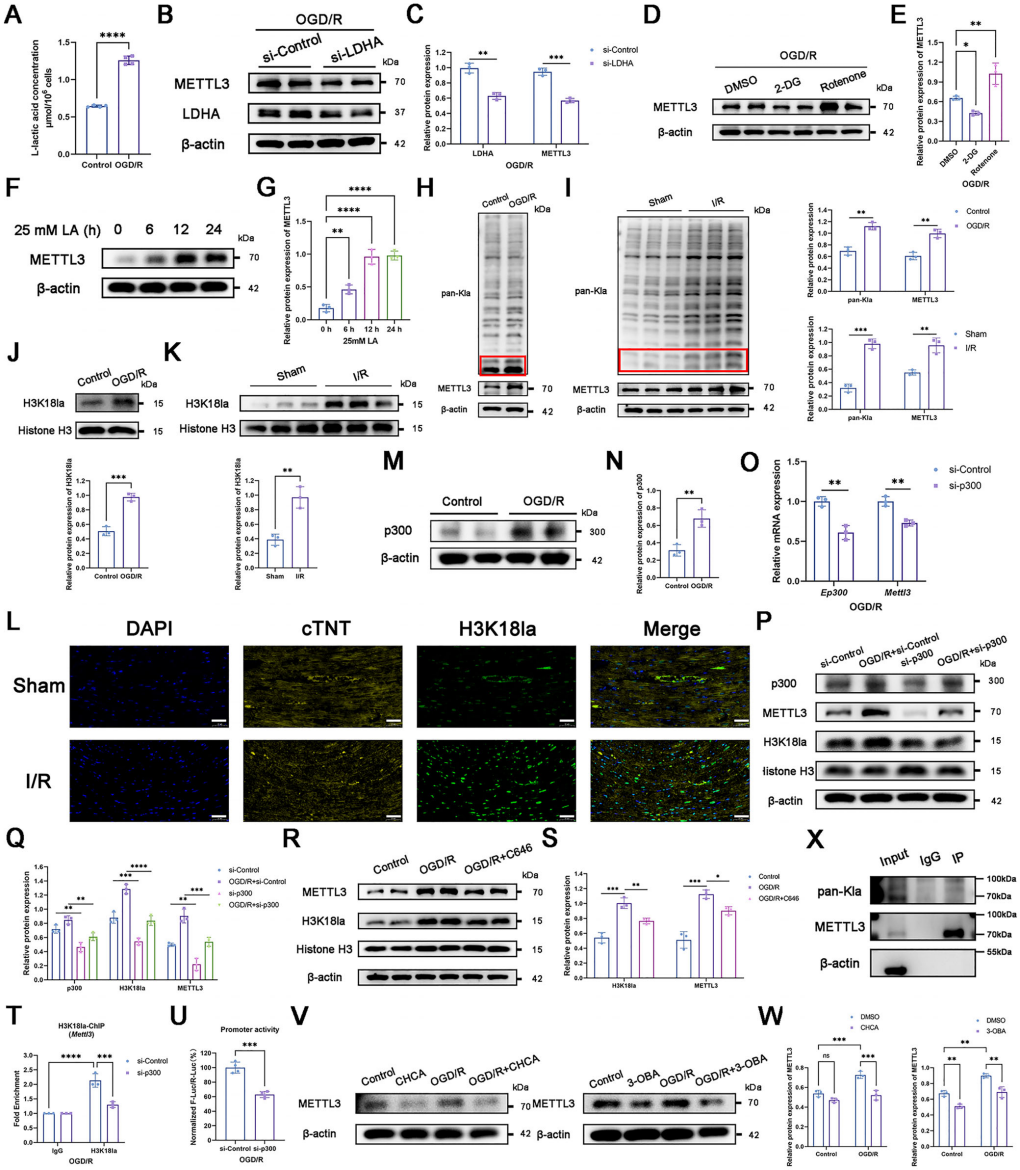
Lactate‐associated p300‐dependent H3K18la contributes to METTL3 transcriptional upregulation following OGD/R and I/R injury. (A) l‐Lactic acid concentrations in NMCMs from the control and OGD/R groups were measured using a lactic acid assay kit (*n* = 4). (B, C) Western blot analysis and quantification of METTL3 protein levels in NMCMs transfected with siRNA targeting LDHA for 48 h under OGD/R conditions (*n* = 3). (D, E) Western blot analysis and quantification of METTL3 protein expression in NMCMs pretreated with 20 mM 2‐DG or 500 nM rotenone for 24 h under OGD/R conditions (*n* = 3). (F, G) Western blot analysis of METTL3 protein expression in NMCMs treated with 25 mM l‐lactic acid for various time periods (*n* = 3). (H, J) Western blot analysis of the indicated proteins in NMCMs following OGD/R treatment (*n* = 3). (I, K) Western blot analysis of the indicated proteins in myocardial tissue subjected to I/R injury (*n* = 3). (L) Representative immunofluorescence images showing H3K18la staining in cardiac sections 24 h after I/R injury (*n* = 5). Scale bar: 50 µm. (M, N) Western blot analysis of p300 expression in NMCMs after OGD/R treatment (*n* = 3). (O) RT‐qPCR analysis of *Ep300* and *Mettl3* mRNA levels in control and p300‐knockdown NMCMs under OGD/R conditions (*n* = 3). (P, Q) Western blot analysis of the indicated proteins in control and p300‐knockdown NMCMs under control or OGD/R conditions (*n* = 3). (R, S) Western blot analysis of the indicated proteins in NMCMs pretreated with or without 100 µM C646 for 24 h under control or OGD/R conditions (*n* = 3). (T) ChIP‐qPCR analysis of H3K18la occupancy at the *Mettl3* promoter in control and p300‐knockdown NMCMs under OGD/R conditions (*n* = 3). (U) Dual‐luciferase reporter assay of the *Mettl3* promoter under OGD/R conditions in NMCMs. Cells were first transfected with si‐Control or si‐p300 for 36 h, then co‐transfected with pGL3‐Mettl3‐luc and pRL‐TK for 36 h, followed by OGD/R treatment. Reporter activity was quantified as the firefly/Renilla luciferase ratio (*n* = 4). (V, W) Western blot analysis of METTL3 protein expression in NMCMs pretreated with 5 mM CHCA or 5 mM 3‐OBA prior to OGD/R treatment (*n* = 3). (X) IP with anti‐METTL3 followed by pan‐Kla immunoblotting to assess whether METTL3 underwent lactylation under OGD/R conditions (*n* = 3). All data are presented as mean ± SD. ns indicates not significant; *p < 0.05; **p < 0.01; ***p < 0.001; ****p < 0.0001.

We next investigated the upstream mechanism of METTL3 induction. Reperfusion‐associated mitochondrial dysfunction and impaired oxidative phosphorylation can promote compensatory glycolysis and lactate accumulation [39, 40, 41]. Consistently, intracellular l‐lactate levels were markedly increased in NMCMs and HL‐1 cells after OGD/R treatment (Figure 2A; Figure S2A).

To determine whether lactate‐associated metabolic remodeling contributes to METTL3 induction, we depleted lactate dehydrogenase A (LDHA), a key enzyme catalyzing lactate production [42, 43]. LDHA knockdown reduced METTL3 protein expression under OGD/R conditions in NMCMs and HL‐1 cells (Figure 2B,C; Figure S2B,C). We then pharmacologically modulated metabolic flux: the glycolysis inhibitor 2‐deoxy‐d‐glucose (2‐DG) suppressed OGD/R‐induced METTL3 expression, whereas the mitochondrial complex I inhibitor rotenone, which promotes compensatory glycolysis and lactate production, further increased METTL3 levels following OGD/R (Figure 2D,E; Figure S2D,E). Moreover, exogenous lactate increased METTL3 protein expression in a time‐dependent manner in both cell types (Figure 2F,G; Figure S2F,G). These findings indicate that OGD/R‐induced METTL3 upregulation is closely linked to lactate‐related metabolic remodeling.

Because lactate‐derived lysine lactylation (Kla) can regulate chromatin and transcription [44], we examined whether lactate accumulation promotes METTL3 expression through enhanced lactylation. pan‐Kla levels increased in OGD/R‐treated NMCMs and HL‐1 cells and in I/R‐injured myocardium, with prominent signals in the histone molecular‐weight range (Figure 2H,I; Figure S2H). We next examined H3K18la, a transcription‐associated histone lactylation mark. H3K18la levels were significantly increased in OGD/R‐treated cardiomyocytes and I/R‐injured myocardium (Figure 2J,K; Figure S2I). Immunofluorescence further confirmed enhanced H3K18la signals with predominant nuclear localization in cardiomyocytes under I/R conditions (Figure 2L), supporting a potential role for H3K18la in METTL3 upregulation following OGD/R and I/R injury.

We next assessed p300, which has been reported to catalyze H3K18 lactylation [44]. We observed a marked increase in p300 protein levels in NMCMs following OGD/R treatment (Figure 2M,N). siRNA‐mediated p300 depletion attenuated OGD/R‐induced METTL3 expression at both the mRNA and protein levels (Figure 2O–Q; Figure S2J–L). Consistently, pharmacological inhibition of p300 with C646 reduced H3K18la levels and concomitantly suppressed METTL3 expression (Figure 2R,S; Figure S2M,N). We next performed H3K18la ChIP‐qPCR. OGD/R increased H3K18la enrichment at the *Mettl3* promoter, whereas p300 knockdown markedly reduced this enrichment (Figure 2T). Consistently, a *Mettl3* promoter‐driven luciferase reporter assay showed that OGD/R enhanced *Mettl3* promoter activity, while p300 knockdown substantially blunted this induction (Figure 2U). These findings support p300‐dependent H3K18la deposition at the *Mettl3* promoter as a contributor to *Mettl3* transcriptional activation.

We also examined the involvement of lactate transport‐ and receptor‐associated pathways. Monocarboxylate transporters (MCTs) mediate lactate transport, whereas G protein‐coupled receptor 81 (GPR81) functions as a lactate‐responsive receptor [45, 46]. Pharmacological inhibition of MCTs with CHCA or GPR81 with 3‐OBA attenuated OGD/R‐induced METTL3 expression (Figure 2V,W), suggesting that lactate transport and receptor‐mediated lactate signaling may also contribute to METTL3 regulation during OGD/R stress.

Finally, because lysine lactylation can occur on non‐histone proteins, we examined whether METTL3 itself was detectably lactylated. Endogenous METTL3 was immunoprecipitated from OGD/R‐treated NMCMs and HL‐1 cells, followed by immunoblotting with a pan‐Kla antibody. No clear pan‐Kla signal was detected in METTL3 immunoprecipitates under our experimental conditions (Figure 2X; Figure S2O). This result suggests that OGD/R‐induced lactylation is more likely to occur on histones and/or other substrates rather than on METTL3 itself.

Collectively, these findings support a lactate‐associated p300‐H3K18la pathway that contributes to METTL3 transcriptional upregulation during OGD/R and I/R injury. Given the complexity of reperfusion‐associated metabolic remodeling, this pathway likely represents an important contributor rather than the sole determinant of METTL3 induction.

### 2.3 METTL3 Promotes PANoptosis‐Related Death Signaling and Multimodal Cell Death Phenotypes in OGD/R‐Treated Cardiomyocytes

**FIGURE 3:**
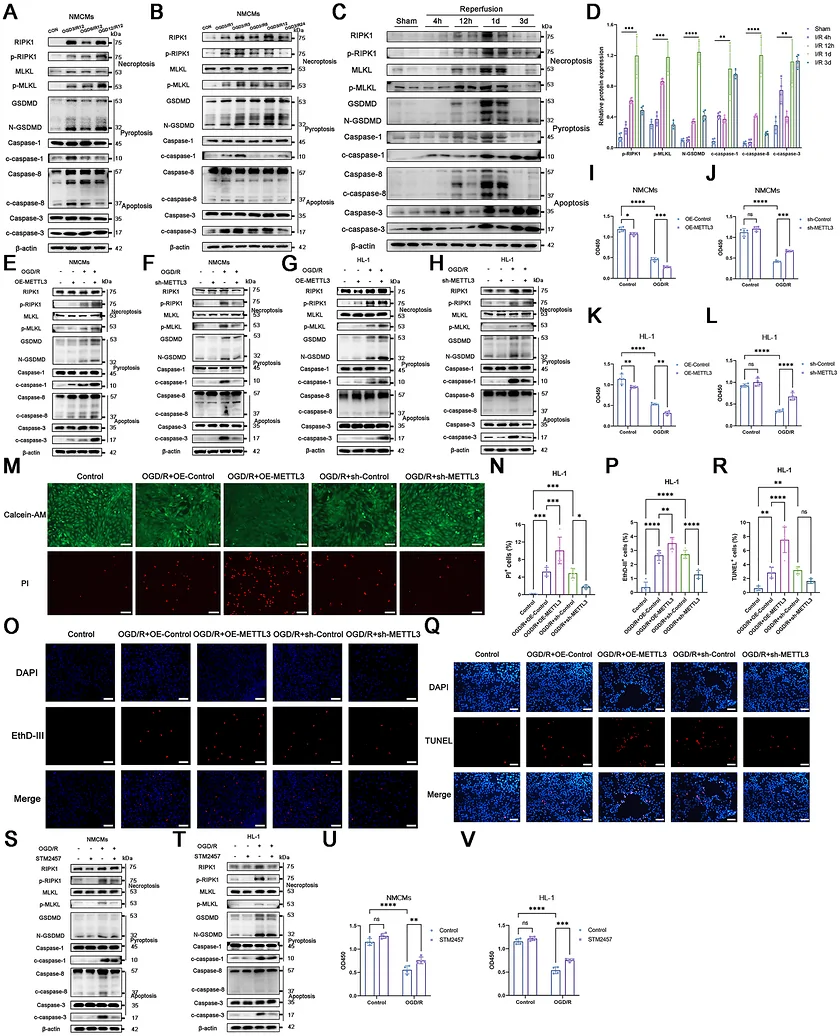
METTL3 promotes PANoptosis‐related death signaling and multimodal cell death phenotypes in OGD/R‐treated cardiomyocytes. (A) Western blot analysis of protein expression in NMCMs exposed to OGD for 0, 3, 6, or 12 h, followed by 12 h of reoxygenation (*n* = 3). (B) Western blot analysis of protein expression in NMCMs subjected to OGD for 3 h, followed by 1, 3, 6, 12, or 24 h of reoxygenation (*n* = 3). (C, D) Western blot analysis of protein expression in myocardial tissue from sham‐operated mice or mice subjected to 45 min of myocardial ischemia followed by 4 h, 12 h, 1 day, or 3 days of reperfusion (*n* = 4). (E, F) Western blot analysis of the indicated proteins in control and METTL3‐overexpressing NMCMs (E) and HL‐1 cells (F) under OGD/R conditions (*n* = 3). (G, H) Western blot analysis of the indicated proteins in control and METTL3‐knockdown NMCMs (G) and HL‐1 cells (H) under OGD/R conditions (*n* = 3). (I–L) Cell viability was assessed using the CCK‐8 assay in NMCMs and HL‐1 cells (*n* = 4). (M, N) Representative images of propidium iodide (PI) staining in HL‐1 cells following the indicated treatments (*n* = 5). Scale bar: 100 µm. (O, P) Representative images of EthD‐III staining in HL‐1 cells following the indicated treatments (*n* = 5). Scale bar: 100 µm. (Q, R) Representative images of TUNEL staining in HL‐1 cells following the indicated treatments (*n* = 5). Scale bar: 100 µm. (S, T) Western blot analysis of the indicated proteins in NMCMs (S) and HL‐1 cells (T) pretreated with DMSO or STM2457 for 24 h, followed by OGD/R treatment (*n* = 3). (U, V) Cell viability was assessed using the CCK‐8 assay in NMCMs and HL‐1 cells (*n* = 4). All data are presented as mean ± SD. ns indicates not significant; *p < 0.05; **p < 0.01; ***p < 0.001; ****p < 0.0001.

PANoptosis is characterized by coordinated activation and crosstalk of apoptosis, pyroptosis, and necroptosis. To evaluate PANoptosis‐related death signaling under OGD/R and I/R conditions, we examined representative apoptosis‐, pyroptosis‐, and necroptosis‐related proteins, including cleaved Caspase‐3 and Caspase‐8, cleaved Caspase‐1 and GSDMD‐N, and p‐MLKL and RIPK1, respectively. These markers increased in parallel in OGD/R‐treated NMCMs and I/R‐injured myocardium during early reoxygenation or reperfusion (Figure 3A–D). Although the magnitude and duration of activation varied among markers, peak induction was observed after 3 h of OGD followed by 3 h of reoxygenation in vitro and at 24 h of reperfusion in vivo, with a partial decline thereafter. This temporal pattern paralleled METTL3 protein induction, suggesting an association between METTL3 upregulation and the coordinated activation of multiple death pathways.

To determine whether METTL3 regulates this response, we generated METTL3‐overexpressing and METTL3‐knockdown models in NMCMs, HL‐1 cells, and AC16 cells. METTL3 overexpression amplified the OGD/R‐induced upregulation of multiple PANoptosis‐related death signaling proteins, whereas METTL3 knockdown markedly attenuated their induction in NMCMs and HL‐1 cells (Figure 3E–H). Consistently, METTL3 overexpression further reduced cell viability, whereas its knockdown partially preserved cell viability (Figure 3I–L). To determine whether pharmacological inhibition recapitulated genetic suppression, cells were pretreated with STM2457, a highly selective METTL3 inhibitor [47]. STM2457 significantly attenuated OGD/R‐induced upregulation of PANoptosis‐related death signaling proteins and concomitantly improved cell viability (Figure 3S–V).

To complement the molecular analyses, we assessed OGD/R‐induced cell death phenotypes using TUNEL, EthD‐III, and PI staining. OGD/R increased TUNEL‐positive cells [48], reflecting DNA fragmentation, as well as EthD‐III‐positive [49] and PI‐positive [50] cells, reflecting compromised membrane integrity associated with lytic cell death (Figure 3M–R; Figure S3A–F). METTL3 overexpression further increased these readouts, whereas knockdown reduced them, consistent with the molecular changes.

To assess crosstalk among these pathways, we selectively inhibited each pathway and examined representative markers. Pharmacological inhibition of apoptosis with the pan‐caspase inhibitor Q‐VD‐OPh reduced cleaved Caspase‐3 but was accompanied by increased p‐MLKL, while GSDMD‐N was largely unchanged (Figure S4A,B). In contrast, inhibition of RIPK3‐dependent necroptosis with GSK‐872 decreased p‐MLKL and concomitantly reduced cleaved Caspase‐3 and GSDMD‐N (Figure S4C,D). Similarly, inhibition of NLRP3 inflammasome signaling with MCC950 reduced GSDMD‐N together with cleaved Caspase‐3 and p‐MLKL (Figure S4E,F).

Collectively, OGD/R and I/R injury induce coordinated multi‐pathway death signaling and multimodal cardiomyocyte death. Genetic and pharmacological evidence further identifies METTL3 as an important promoter of these PANoptosis‐related responses under reperfusion‐associated stress.

### 2.4 METTL3‐Regulated ZBP1 Promotes PANoptosis‐Related Component Interactions and Death Signaling in Cardiomyocytes Under OGD/R and I/R Stress

**FIGURE 4:**
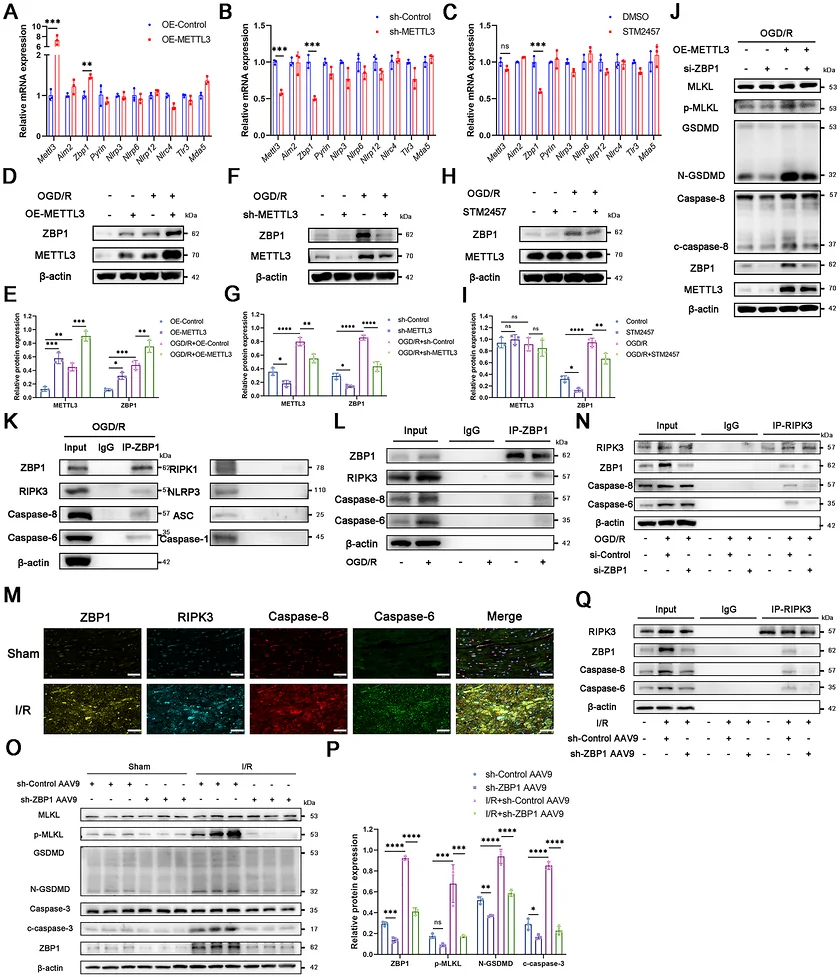
METTL3‐regulated ZBP1 promotes PANoptosis‐related component interactions and death signaling in cardiomyocytes under OGD/R and I/R stress. (A) RT‐qPCR analysis of *Mettl3* and PANoptosis‐related sensor mRNA levels in control and METTL3‐overexpressing NMCMs under OGD/R conditions (*n* = 3). (B) RT‐qPCR analysis of *Mettl3* and PANoptosis‐related sensor mRNA levels in control and METTL3‐knockdown NMCMs under OGD/R conditions (*n* = 3). (C) RT‐qPCR analysis of *Mettl3* and PANoptosis‐related sensor mRNA levels in NMCMs treated with DMSO or STM2457 under OGD/R conditions (*n* = 3). (D–I) Western blot analysis of ZBP1 protein expression in NMCMs under the conditions described in (A–C) (*n* = 3). (J) Western blot analysis of PANoptosis‐related death signaling protein levels in control and METTL3‐overexpressing NMCMs following ZBP1 knockdown under OGD/R conditions (*n* = 3). (K) Co‐IP analysis of ZBP1 and PANoptosis‐related components in OGD/R‐treated NMCMs (*n* = 3). (L) Co‐IP validation of the interactions between ZBP1 and PANoptosis‐related components in HL‐1 cells under control and OGD/R conditions (*n* = 3). (M) Representative immunofluorescence co‐localization staining of ZBP1, RIPK3, Caspase‐8, and Caspase‐6 in heart sections from Sham and I/R mice (*n* = 3). Scale bar: 50 µm. (N) RIPK3 Co‐IP analysis of ZBP1, Caspase‐8, and Caspase‐6 in OGD/R‐treated NMCMs transfected with si‐Control or si‐ZBP1 (*n* = 3). (O–P) Western blot analysis of PANoptosis‐related death signaling protein levels in myocardial tissue from control and cardiomyocyte‐specific ZBP1‐knockdown mice at 1 day post‐I/R injury (*n* = 3). (Q) RIPK3 Co‐IP analysis of ZBP1, Caspase‐8, and Caspase‐6 in myocardial tissue from control and cardiomyocyte‐specific ZBP1‐knockdown mice at 1 day post‐I/R injury (*n* = 3). All data are presented as mean ± SD. ns indicates not significant; *p < 0.05; **p < 0.01; ***p < 0.001; ****p < 0.0001.

PANoptosis can be initiated by distinct innate immune sensors and coordinated through multiprotein signaling platforms [27]. Guided by previous reports [51], we screened several candidate innate immune sensors, including ZBP1, AIM2, NLRP12, and others, for responsiveness to METTL3 manipulation under OGD/R conditions. Among the candidates tested, *Zbp1* mRNA showed the most consistent and robust response to METTL3 regulation: METTL3 overexpression increased *Zbp1* mRNA expression, whereas METTL3 knockdown or STM2457 treatment reduced its expression in OGD/R‐treated NMCMs (Figure 4A–C). This pattern was further confirmed at the protein level, with ZBP1 abundance changing concordantly with METTL3 manipulation (Figure 4D–I). Moreover, OGD/R induced the activation of multiple PANoptosis‐related death signaling proteins, whereas ZBP1 knockdown substantially attenuated these inductions (Figure 4J), supporting ZBP1 as a METTL3‐responsive regulator of PANoptosis‐related death signaling in cardiomyocytes.

We next examined whether ZBP1 participates in interactions among PANoptosis‐related components in cardiomyocytes. Given that ZBP1‐containing PANoptosome complexes have been reported in infectious and inflammatory models, we tested whether a similar protein interaction pattern could be detected in cardiomyocytes under OGD/R conditions. Co‐IP analysis in OGD/R‐treated NMCMs showed that ZBP1 associated with RIPK3, Caspase‐8, and Caspase‐6, whereas interactions with RIPK1, NLRP3, ASC, or Caspase‐1 were not robustly detected under our experimental conditions (Figure 4K). Similar results were obtained in HL‐1 cells, in which OGD/R strengthened these associations (Figure 4L). Quadruple immunofluorescence staining showed minimal overlap of ZBP1, RIPK3, Caspase‐8, and Caspase‐6 in Sham hearts but pronounced co‐localization in I/R‐injured myocardium (Figure 4M). This increased spatial proximity of these molecules was consistent with activation of PANoptosis‐related death signaling in vivo, suggesting coordinated spatial organization of these components during myocardial I/R injury.

To determine whether these interactions depend on ZBP1, we performed RIPK3‐based Co‐IP following ZBP1 knockdown. OGD/R promoted the association of RIPK3 with ZBP1, Caspase‐8, and Caspase‐6, whereas ZBP1 depletion markedly weakened these interactions (Figure 4N), indicating that ZBP1 is required for the robust association of these PANoptosis‐related components in OGD/R‐treated cardiomyocytes.

We further validated these findings in vivo using AAV9‐mediated cardiomyocyte‐specific ZBP1 knockdown. At 1 day after I/R injury, ZBP1 depletion reduced PANoptosis‐related death signaling in myocardial tissue (Figure 4O,P) and weakened the associations of RIPK3 with ZBP1, Caspase‐8, and Caspase‐6 (Figure 4Q). These in vivo loss‐of‐function data support an important role for ZBP1 in coordinating these component interactions and promoting PANoptosis‐related death signaling during myocardial I/R injury.

Collectively, ZBP1 is required for robust interactions among these components and promotes PANoptosis‐related death signaling during myocardial I/R injury, although the precise composition and dynamics of the complex require further investigation.

### 2.5 Cardiomyocyte‐Specific METTL3 Knockout Attenuates I/R‐Induced PANoptosis‐Related Death Signaling and Alleviates Cardiac Dysfunction and Adverse Remodeling

**FIGURE 5:**
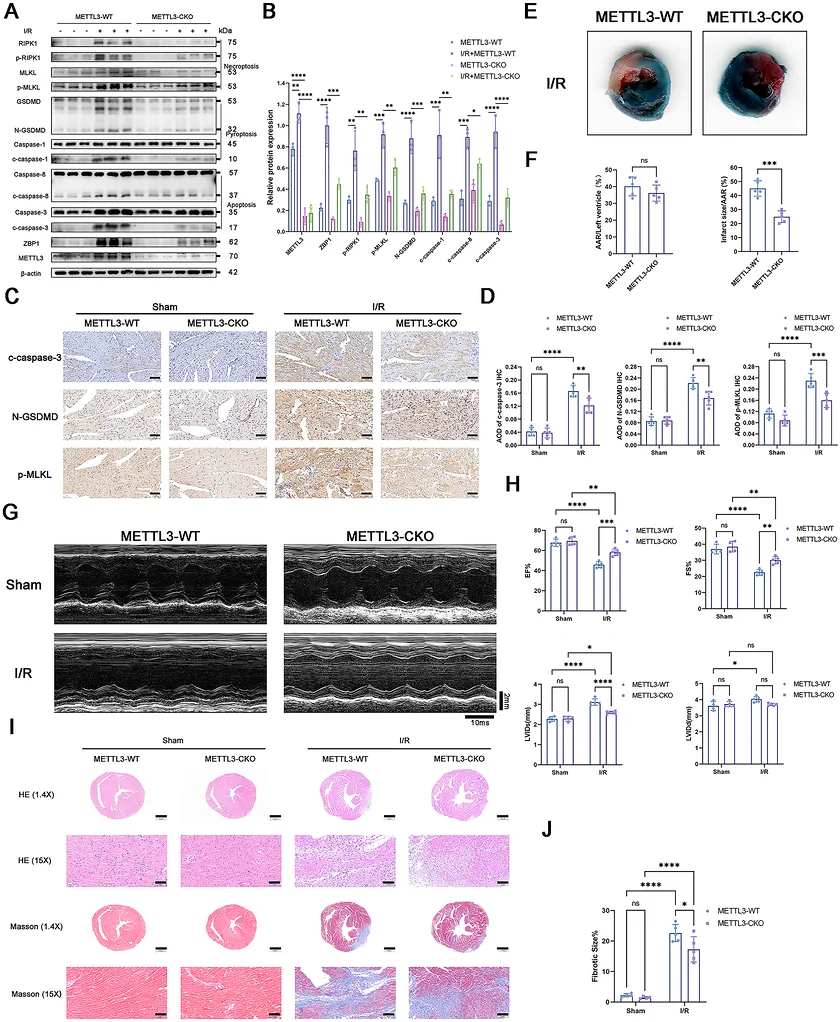
Cardiomyocyte‐specific METTL3 knockout attenuates I/R‐induced PANoptosis‐related death signaling and alleviates cardiac dysfunction and adverse remodeling. (A, B) Western blot analysis of the indicated proteins in myocardial tissue from METTL3‐WT and METTL3‐CKO mice at 1 day post‐I/R injury (*n* = 3). (C, D) Immunohistochemical staining of cardiac sections from METTL3‐WT and METTL3‐CKO mice at 1 day post‐I/R injury (*n* = 5). Scale bar: 100 µm. (E, F) Representative images of 2,3,5‐triphenyltetrazolium chloride (TTC)‐stained transverse sections of hearts after Evans blue perfusion. The area at risk (AAR) and infarct size were measured 24 h after I/R injury (*n* = 5). (G) Representative M‐mode echocardiography images at 3 weeks post‐I/R injury (*n* = 4–5). (H) Quantitative analysis of echocardiographic parameters. EF, ejection fraction; FS, fractional shortening; LVIDd, left ventricular internal diameter at end‐diastolic phase; LVIDs, left ventricular internal diameter at end‐systolic phase (*n* = 4–5). (I) H&E staining and Masson's trichrome staining of cardiac sections at 3 weeks post‐I/R injury (*n* = 4–5). Scale bar: 100 or 1000 µm. (J) Scar size quantified as the percentage of the left ventricular area (*n* = 4–5). All data are presented as mean ± SD. ns indicates not significant; *p < 0.05; **p < 0.01; ***p < 0.001; ****p < 0.0001.

To investigate the role of METTL3 in cardiomyocytes in vivo, we generated cardiomyocyte‐specific, tamoxifen‐inducible conditional knockout mice (*Mettl3^fl/fl^; Myh6‐Cre^ERT2+^*, METTL3‐CKO) and littermate control mice (*Mettl3^fl/fl^; Myh6‐Cre^ERT2−^*, METTL3‐WT). At 24 h after I/R injury, Western blotting confirmed efficient METTL3 deletion and reduced expression of multiple PANoptosis‐related death signaling proteins in METTL3‐CKO hearts (Figure 5A,B). Immunohistochemical staining of cardiac sections showed significantly lower average optical densities of the indicated markers in METTL3‐CKO hearts than in METTL3‐WT hearts (Figure 5C,D). Evans blue/TTC staining at 1 day after I/R injury showed a smaller infarct size in METTL3‐CKO mice, with corresponding quantification of the area at risk and infarct area (Figure 5E,F). Echocardiographic assessment at 3 weeks after I/R injury showed better preservation of cardiac function in METTL3‐CKO mice, as reflected by increased EF and FS and reduced LVIDd and LVIDs (Figure 5G,H). Histological analyses showed that METTL3‐CKO hearts exhibited improved myocardial architecture, reduced myofiber disarray and inflammatory infiltration on H&E staining, and decreased collagen deposition and scar formation on Masson's trichrome staining (Figure 5I,J). Collectively, these data indicate that cardiomyocyte‐specific METTL3 deletion attenuates I/R‐induced PANoptosis‐related death signaling and protects against subsequent cardiac dysfunction and adverse remodeling.

**FIGURE 6:**
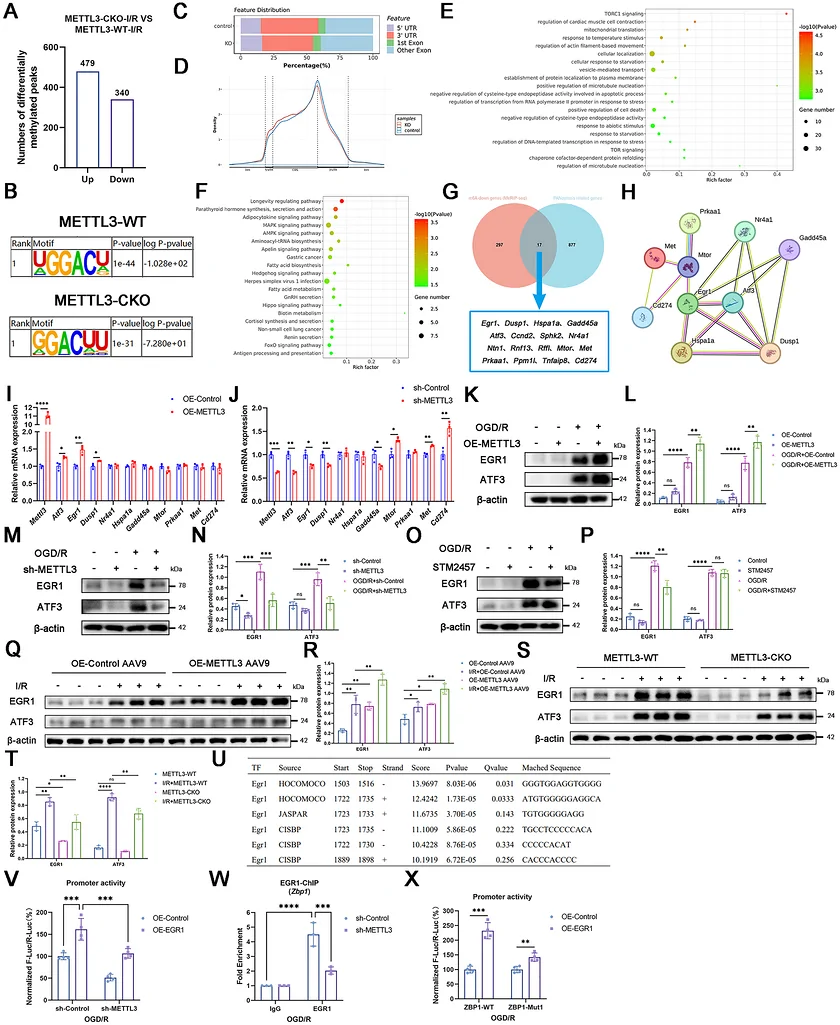
METTL3‐dependent m^6^A regulation of EGR1 promotes EGR1‐mediated transcriptional activation of *Zbp1*. (A) Differential m^6^A peak analysis between METTL3‐CKO and METTL3‐WT groups based on MeRIP‐seq. (B) Representative motif analysis of differential m^6^A peaks in myocardial tissue from METTL3‐WT and METTL3‐CKO mice. (C, D) Distribution of m^6^A peak locations in myocardial tissue from METTL3‐WT and METTL3‐CKO mice. (E) Gene ontology (GO) enrichment analysis of genes with reduced m^6^A peaks in METTL3‐CKO compared with METTL3‐WT groups based on MeRIP‐seq data. (F) Kyoto Encyclopedia of Genes and Genomes (KEGG) pathway enrichment analysis of genes with reduced m^6^A peaks in METTL3‐CKO compared with METTL3‐WT groups based on MeRIP‐seq data. (G) Venn diagram showing the overlap between genes with reduced m^6^A modification and PANoptosis‐related genes. (H) Protein–protein interaction (PPI) network of the 10 overlapping candidate genes. (I, J) mRNA expression levels of *Mettl3* and candidate genes in control and METTL3‐overexpressing (I) or METTL3‐knockdown (J) NMCMs under OGD/R conditions (*n* = 3). (K–T) Western blot analysis of EGR1 and ATF3 protein levels in NMCMs following METTL3 overexpression (K, L), METTL3 knockdown (M, N), or STM2457 treatment (O, P) under OGD/R conditions; and in myocardial tissue from OE‐Control and OE‐METTL3 mice (Q, R) or from METTL3‐WT and METTL3‐CKO mice (S, T) at 1 day post‐I/R injury (*n* = 3). (U) Candidate EGR1‐binding sites in the *Zbp1* promoter region (−2000 to +100 bp) predicted using multiple databases. (V) Construction of a luciferase reporter plasmid containing the truncated *Zbp1* promoter sequence (−600 to +100 bp), followed by dual‐luciferase reporter assay (*n* = 4). (W) ChIP‐qPCR analysis of EGR1 enrichment at the *Zbp1* promoter region in control and METTL3‐knockdown NMCMs under OGD/R conditions (*n* = 3). (X) Based on multi‐database prediction results, a mutant reporter plasmid targeting the candidate EGR1‐binding site at −279 to −266 bp of the *Zbp1* promoter was constructed and subjected to dual‐luciferase reporter assay (*n* = 4). All data are presented as mean ± SD. ns indicates not significant; *p < 0.05; **p < 0.01; ***p < 0.001; ****p < 0.0001.

### 2.6 Cardiomyocyte‐Specific METTL3 Overexpression Exacerbates I/R‐Induced PANoptosis‐Related Death Signaling and Aggravates Cardiac Dysfunction and Adverse Remodeling

To further validate the role of METTL3 in vivo, we established a cardiomyocyte‐specific METTL3 overexpression model by intravenous injection of AAV9‐METTL3. Three weeks later, mice were subjected to myocardial I/R injury. Western blot analysis confirmed increased METTL3 protein expression and showed a parallel upregulation of multiple PANoptosis‐related death signaling proteins in METTL3‐overexpressing hearts (Figure S5A,B). Immunohistochemical staining yielded consistent results, with enhanced staining intensity for the indicated markers after I/R injury (Figure S5C,D). METTL3 overexpression also increased myocardial infarct size, aggravated post‐I/R cardiac dysfunction, and exacerbated myocardial fibrosis and scar formation (Figure S5E–J). These findings further support a pathogenic role for METTL3 in myocardial I/R injury and subsequent adverse remodeling.

### 2.7 METTL3‐Dependent m^6^A Regulation of EGR1 Promotes EGR1‐Mediated Transcriptional Activation of *Zbp1*

To identify downstream m^6^A‐modified targets of METTL3, we performed m^6^A RNA immunoprecipitation sequencing (MeRIP‐seq) using infarct‐border‐zone myocardium from METTL3‐WT and METTL3‐CKO mice after I/R injury. With thresholds of *p* < 0.05 and |log_2_FC| > 1, we identified 479 hypermethylated and 340 hypomethylated m^6^A peaks (Figure 6A). These m^6^A peaks were enriched in the RRACH motif and were predominantly located near the CDS‐3′UTR junctions (Figure 6B–D). Functional enrichment analysis of hypomethylated transcripts highlighted stress‐adaptive processes, including TORC1/TOR signaling, cellular stress responses, cardiac contraction, and mitochondrial translation (Figure 6E). Cell death‐related GO terms were also enriched, suggesting that METTL3‐dependent m^6^A remodeling may influence death‐associated regulatory programs. KEGG analysis further identified AMPK, MAPK, FoxO, and Hippo signaling, along with pathways involved in metabolic reprogramming and immune regulation, including antigen processing and presentation (Figure 6F). We then intersected the hypomethylated transcripts with 902 nonredundant PANoptosis‐related genes compiled from GeneCards, including 881 apoptosis‐related, 12 necroptosis‐related, and 29 pyroptosis‐related entries, with overlap among the three categories (relevance score > 3). This analysis identified 17 candidates, 10 of which were functionally connected in protein–protein interaction analysis (Figure 6G,H).

Bidirectional METTL3 manipulation in OGD/R‐treated NMCMs and AC16 cells identified *Egr1/EGR1* and *Atf3/ATF3* as the only transcripts consistently responsive across both models (Figure 6I,J; Figure S6A,B). METTL3 also modulated EGR1 and ATF3 protein abundance. However, STM2457 markedly reduced EGR1 protein levels while leaving ATF3 largely unchanged (Figure 6K–P; Figure S6C–N), and similar patterns were observed in I/R‐injured myocardium (Figure 6Q–T). Together, these results suggest that ATF3 may be regulated by METTL3 through a mechanism less dependent on its catalytic activity, whereas EGR1 shows a methyltransferase activity‐dependent response consistent with m^6^A‐mediated regulation.

Given that EGR1 is a transcription factor, we next investigated whether it links METTL3 signaling to *Zbp1* transcriptional regulation. Binding‐site prediction using JASPAR, HOCOMOCO, and CIS‐BP identified several putative EGR1‐binding motifs within the proximal *Zbp1* promoter. Among them, the motif located at approximately −279 to −266 bp relative to the transcription start site showed a high prediction score and was consistently predicted across the three databases (Figure 6U). We therefore cloned the −600 to +100 bp *Zbp1* promoter fragment into a luciferase reporter vector. Dual‐luciferase reporter assays showed that EGR1 enhanced *Zbp1* promoter activity, whereas METTL3 knockdown reduced both basal and EGR1‐induced promoter activity (Figure 6V). ChIP‐qPCR further confirmed EGR1 enrichment at the *Zbp1* promoter, which was reduced by METTL3 knockdown (Figure 6W). Moreover, mutation of the −279 to −266 bp motif markedly attenuated EGR1‐induced promoter activation (Figure 6X), identifying this motif as an important functional EGR1‐responsive element within the *Zbp1* promoter.

Together, these findings support a direct role for EGR1 in the transcriptional activation of *Zbp1*. Combined with the upstream regulation of EGR1 by METTL3, they define a METTL3‐EGR1‐ZBP1 regulatory axis that contributes to ZBP1‐associated PANoptosis‐related death signaling in cardiomyocytes.

### 2.8 The METTL3‐EGR1 Axis Promotes ZBP1‐Associated PANoptosis‐Related Death Signaling In Vitro and In Vivo

To determine whether EGR1 mediates METTL3‐dependent regulation of PANoptosis‐related death signaling in cardiomyocytes under OGD/R conditions, we performed rescue experiments. Consistent with the effects of METTL3, EGR1 bidirectionally regulated multiple PANoptosis‐related death signaling proteins. EGR1 overexpression increased multiple PANoptosis‐related death signaling proteins, whereas EGR1 knockdown reduced their induction under OGD/R stress (Figure S7A,B). Moreover, ZBP1 knockdown markedly attenuated the EGR1‐induced elevation of these markers (Figure S7C), indicating that EGR1‐driven amplification of PANoptosis‐related death signaling is at least partly dependent on ZBP1, supporting a functional EGR1‐ZBP1 link under OGD/R stress.

In METTL3‐knockdown cells, EGR1 overexpression partially restored ZBP1 and PANoptosis‐related death signaling protein levels and reduced cell viability (Figure S7D,E). Conversely, in METTL3‐overexpressing cells, EGR1 knockdown attenuated the effects of METTL3 overexpression, reducing PANoptosis‐related death signaling and improving cell viability (Figure S7F,G). Calcein‐AM/PI, EthD‐III, and TUNEL staining consistently showed that EGR1 overexpression partially reversed the reduction in death‐positive cells caused by METTL3 knockdown (Figure S7H–M).

To validate the role of the METTL3‐EGR1 axis in vivo, AAV9‐EGR1 was delivered to METTL3‐CKO mice before I/R injury. EGR1 restoration increased ZBP1 expression and PANoptosis‐related death signaling proteins, as shown by Western blotting and immunohistochemistry (Figure S8A–D). It also partially reversed the cardioprotective effects of METTL3 deletion, resulting in larger infarcts, worse cardiac function, and greater myocardial fibrosis (Figure S8E–J).

Collectively, these in vitro and in vivo rescue experiments indicate that EGR1 acts downstream of METTL3 to promote ZBP1‐associated PANoptosis‐related death signaling in cardiomyocytes.

### 2.9 METTL3 Enhances *Egr1* mRNA Stability and Translational Efficiency via m^6^A Modification

MeRIP‐seq identified three m^6^A peaks within *Egr1* that were reduced in METTL3‐deficient myocardium (Figure S9A). SRAMP predicted five candidate m^6^A sites within these regions, including four in the CDS and one in the 3′UTR (Figure S9B). RIP‐qPCR confirmed METTL3 binding to *Egr1* mRNA, which was enhanced by OGD/R or METTL3 overexpression (Figure S9C,D), supporting *Egr1* as a METTL3‐dependent m^6^A target.

We next examined whether METTL3 regulates EGR1 transcriptionally or posttranscriptionally. METTL3 knockdown reduced mature *Egr1* mRNA without affecting *Egr1* promoter activity or pre‐mRNA abundance (Figure S9E,F). Actinomycin D chase assays showed accelerated decay of mature *Egr1* mRNA, but not pre‐mRNA, following METTL3 depletion (Figure S9G,H). EGR1 protein half‐life and subcellular localization were not significantly affected by METTL3 knockdown (Figure S9I,J,M,N). Polysome profiling showed that METTL3 knockdown reduced *Egr1* mRNA abundance in polysome fractions (Figure S9K,L). These results indicate that METTL3 promotes EGR1 expression mainly through posttranscriptional regulation by stabilizing mature *Egr1* mRNA and enhancing its translational activity.

**FIGURE 7:**
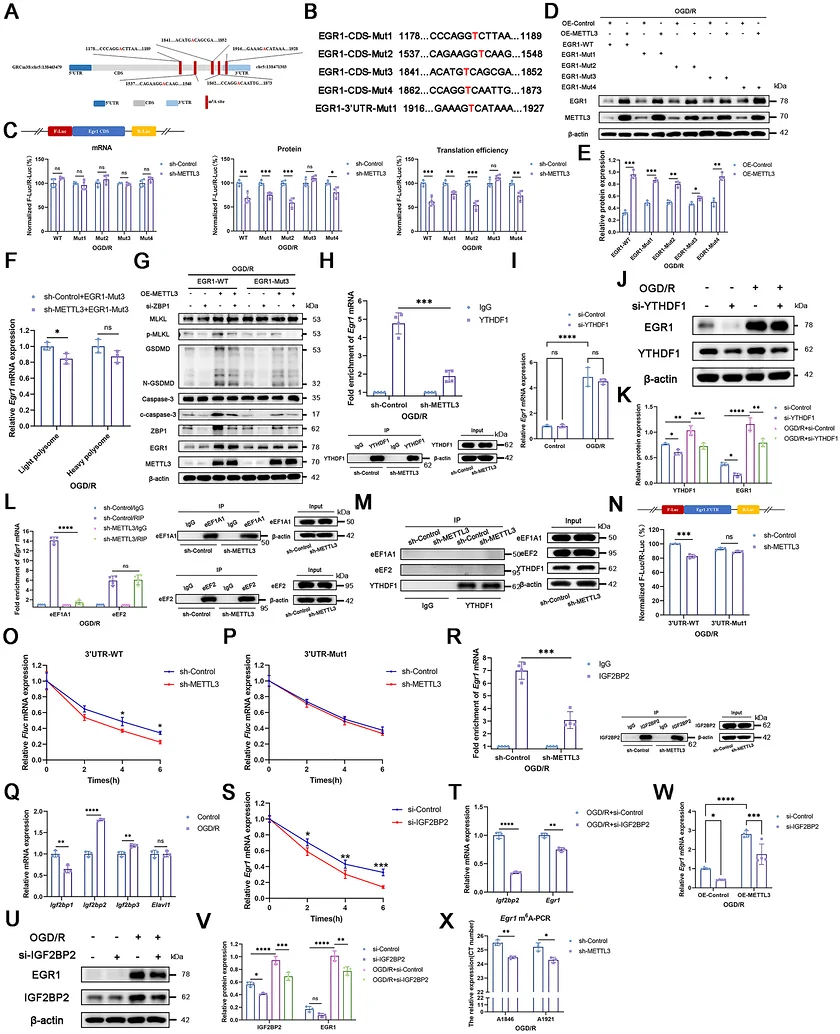
METTL3‐mediated m^6^A modification of *Egr1* mRNA promotes YTHDF1‐associated translational upregulation and IGF2BP2‐dependent mRNA stabilization. (A) Schematic representation of the predicted m^6^A motif distribution along the *Egr1* mRNA transcript. (B) Schematic representation of pmirGLO vector constructs with GGAC/GAC‐to‐GGTC/GTC mutations to investigate the role of m^6^A modifications in the CDS or 3'UTR in regulating EGR1 expression. (C) Control and METTL3‐knockdown HL‐1 cells were transfected with pmirGLO‐EGR1‐CDS‐WT or pmirGLO‐EGR1‐CDS‐Mut1/2/3/4 plasmids for 36 h and then subjected to OGD/R treatment. Reporter activity was expressed as the ratio of firefly luciferase (Fluc) to Renilla luciferase (Rluc). *Fluc* and *Rluc* mRNA levels were measured by RT‐qPCR, and translational efficiency was calculated as the ratio of normalized luciferase activity to the corresponding mRNA level (*n* = 4). (D, E) Western blot analysis and quantification of EGR1 protein in METTL3‐overexpressing HL‐1 cells transfected with pcDNA3.1‐EGR1‐WT or pcDNA3.1‐EGR1‐Mut1/2/3/4 plasmids under OGD/R conditions (*n* = 3). (F) Distribution of pcDNA3.1‐EGR1‐Mut3‐derived *Egr1* mRNA across light and heavy polysome fractions in control and METTL3‐knockdown HL‐1 cells under OGD/R conditions (*n* = 3). (G) Western blot analysis of the indicated proteins in HL‐1 cells subjected to OGD/R under three‐factor intervention, including METTL3 overexpression, ZBP1 knockdown, and pcDNA3.1‐EGR1‐WT or Mut3 expression (*n* = 3). (H) RIP‐qPCR analysis of *Egr1* mRNA binding to YTHDF1 in control and METTL3‐knockdown HL‐1 cells under OGD/R conditions. Enrichment was normalized to input (*n* = 4). (I) RT‐qPCR analysis of *Ythdf1* and *Egr1* mRNA levels in control and YTHDF1‐knockdown HL‐1 cells under OGD/R conditions (*n* = 3). (J, K) Western blot analysis of the indicated proteins in control and YTHDF1‐knockdown HL‐1 cells under OGD/R conditions (*n* = 3). (L) RIP‐qPCR analysis of *Egr1* mRNA binding to eEF1A1 or eEF2 in control and METTL3‐knockdown HL‐1 cells under OGD/R conditions (*n* = 4). (M) Co‐IP analysis of interactions between YTHDF1 and eEF1A1 or eEF2 in control and METTL3‐knockdown HL‐1 cells under OGD/R conditions (*n* = 3). (N) Luciferase reporter assay in control and METTL3‐knockdown HL‐1 cells transfected with pmirGLO‐EGR1‐3′UTR‐WT or pmirGLO‐EGR1‐3′UTR‐Mut1 under OGD/R conditions (*n* = 3). (O, P) RT‐qPCR analysis of *Fluc* mRNA levels in control and METTL3‐knockdown cells transfected with pmirGLO‐EGR1‐3′UTR‐WT or pmirGLO‐EGR1‐3′UTR‐Mut1 for 36 h, followed by actinomycin D (5 µg/mL) treatment for the indicated times (*n* = 3). (Q) RT‐qPCR analysis of mRNA levels of *Igf2bp1*, *Igf2bp2*, *Igf2bp3*, and *Elavl1* in HL‐1 cells under OGD/R conditions (*n* = 3). (R) RIP‐qPCR analysis of the interaction of *Egr1* mRNA with IGF2BP2 in control and METTL3‐knockdown cells under OGD/R conditions. Enrichment of *Egr1* mRNA was measured by qPCR and normalized to input (*n* = 4). (S) *Egr1* mRNA stability analysis in control and IGF2BP2‐knockdown HL‐1 cells treated with actinomycin D (5 µg/mL) for the indicated times (*n* = 3). (T) RT‐qPCR analysis of *Igf2bp2* and *Egr1* mRNA levels in control and IGF2BP2‐knockdown HL‐1 cells under OGD/R conditions (*n* = 3). (U, V) Western blot analysis of IGF2BP2 and EGR1 protein levels in control and IGF2BP2‐knockdown HL‐1 cells under OGD/R conditions (*n* = 3). (W) RT‐qPCR analysis of *Egr1* mRNA levels in METTL3‐overexpressing HL‐1 cells with or without IGF2BP2 knockdown under OGD/R conditions (*n* = 4). (X) SELECT assay for site‐specific detection and comparison of m^6^A modification at the A1846 and A1921 sites of *Egr1* mRNA in control and METTL3‐knockdown HL‐1 cells under OGD/R conditions (*n* = 3). All data are presented as mean ± SD. ns indicates not significant; *p < 0.05; **p < 0.01; ***p < 0.001; ****p < 0.0001.

Because the effects of m^6^A modification may depend on transcript position, with 5′UTR m^6^A facilitating cap‐independent translation initiation [52], CDS m^6^A regulating translation [53], and 3′UTR m^6^A influencing mRNA stability [54], we first examined candidate m^6^A sites within the *Egr1* CDS. A wild‐type luciferase reporter and four reporters carrying individual GGAC‐to‐GGTC substitutions were generated (Figure 7A,B). Translational efficiency was calculated by normalizing reporter activity to reporter mRNA abundance. Among the candidate sites, mutation of A1846 markedly attenuated METTL3‐dependent changes in translational efficiency (Figure 7C). To validate this site in full‐length EGR1, the same mutations were introduced into *Egr1* expression constructs. Under METTL3 overexpression, the A1846‐mutant construct (EGR1‐Mut3) showed minimal protein induction compared with the wild‐type and other mutant constructs (Figure 7D,E). Moreover, the polysome association of A1846‐mutant *Egr1* mRNA was minimally affected by METTL3 knockdown (Figure 7F). Functionally, METTL3 overexpression increased ZBP1 and PANoptosis‐related death signaling proteins in cells expressing EGR1‐WT, whereas these effects were substantially attenuated in cells expressing EGR1‐Mut3 and were further reduced by ZBP1 knockdown (Figure 7G). These findings identify A1846 within exon 2 of *Egr1* as a functional METTL3‐dependent m^6^A site that promotes EGR1 translation and downstream ZBP1‐associated death signaling.

Because YTHDF1 promotes the translation of m^6^A‐modified transcripts [53], we next examined its involvement in *Egr1* mRNA regulation. RIP‐qPCR showed that YTHDF1 bound *Egr1* mRNA under OGD/R conditions, whereas METTL3 knockdown weakened this interaction (Figure 7H). YTHDF1 depletion reduced EGR1 protein expression without appreciably affecting *Egr1* mRNA abundance (Figure 7I–K), supporting its involvement in METTL3‐dependent translational regulation.

We next assessed the involvement of the translation elongation factors eEF1A1 and eEF2 [55, 56]. *Egr1* mRNA associated with both proteins under OGD/R conditions, but METTL3 knockdown selectively reduced its association with eEF1A1 (Figure 7L). However, Co‐IP did not detect stable interactions between YTHDF1 and either eEF1A1 or eEF2 (Figure 7M). Thus, although YTHDF1 contributes to METTL3‐dependent *Egr1* translation, the current data do not support direct recruitment of eEF1A1 by YTHDF1. The altered association between eEF1A1 and *Egr1* mRNA should therefore be considered an exploratory observation that may reflect broader METTL3/m^6^A‐dependent changes in translational regulation.

Because METTL3 also enhanced the stability of mature *Egr1* mRNA, we next examined the predicted A1921 m^6^A site within the *Egr1* 3′UTR. Wild‐type or A1921‐mutant *Egr1* 3′UTR sequences were cloned downstream of the firefly luciferase coding region (Figure 7A,B). A1921 mutation abolished METTL3‐dependent regulation of luciferase activity (Figure 7N). Actinomycin D chase assays further showed that METTL3 knockdown accelerated the decay of reporter mRNA containing the wild‐type 3′UTR but had little effect on the A1921‐mutant transcript (Figure 7O,P). These findings identify A1921 as a functional m^6^A site required for METTL3‐dependent stabilization of *Egr1* mRNA.

Given that m^6^A readers such as IGF2BPs and HuR can stabilize target transcripts through direct binding [13], we next investigated the m^6^A reader responsible for this effect. IGF2BP2 expression was increased after OGD/R treatment (Figure 7Q). RIP‐qPCR revealed that IGF2BP2 bound *Egr1* mRNA and that METTL3 knockdown weakened this interaction (Figure 7R). Actinomycin D chase assays further showed that IGF2BP2 knockdown accelerated *Egr1* mRNA decay (Figure 7S), reduced both *Egr1* mRNA and EGR1 protein levels (Figure 7T–V), and reversed METTL3‐induced *Egr1* mRNA upregulation (Figure 7W). These results indicate that IGF2BP2 mediates METTL3‐dependent stabilization of *Egr1* mRNA.

Finally, site‐specific m^6^A modification was assessed using SELECT, which quantifies ligation and extension products by RT‐qPCR. METTL3 knockdown reduced relative m^6^A levels at both A1846 and A1921 in HL‐1 cells, supporting these sites as key METTL3‐dependent regulatory sites (Figure 7X).

Collectively, METTL3 promotes EGR1 expression through two complementary mechanisms: m^6^A modification at A1846 within the CDS supports YTHDF1‐associated translational regulation, whereas modification at A1921 within the 3′UTR promotes IGF2BP2‐dependent mRNA stabilization.

### 2.10 Pharmacological Inhibition of METTL3 With STM2457 Attenuates I/R‐Induced Myocardial Injury and Adverse Cardiac Remodeling

**FIGURE 8:**
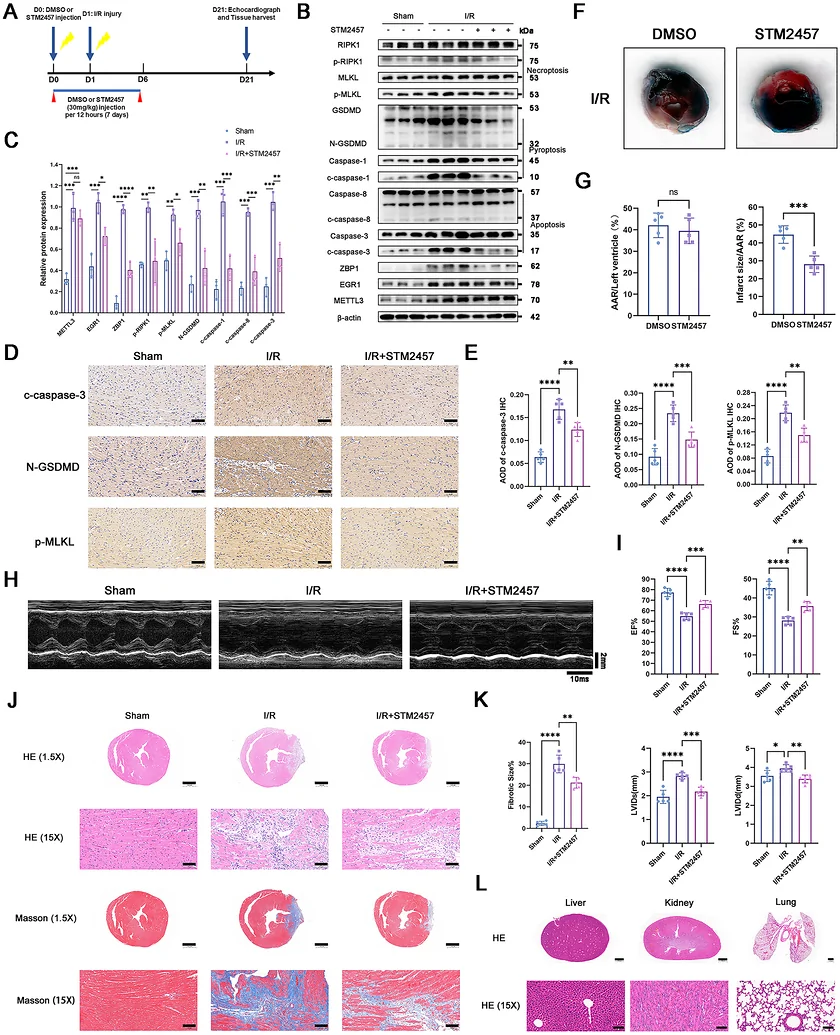
Pharmacological inhibition of METTL3 with STM2457 attenuates I/R‐induced myocardial injury and adverse cardiac remodeling. (A) Schematic representation showing that mice were administered the METTL3 inhibitor STM2457 intraperitoneally every 12 h, starting one day prior to I/R surgery and continuing for six consecutive days after surgery. (B, C) Western blot analysis of the indicated proteins in myocardial tissue from control and STM2457‐treated mice at 1 day post‐I/R injury (*n* = 3). (D, E) Immunohistochemical staining of cardiac sections from control and STM2457‐treated mice at 1 day post‐I/R injury (*n* = 5). Scale bar: 100 µm. (F, G) Representative images of TTC‐stained transverse sections of hearts after Evans blue perfusion. The AAR and infarct size were measured 24 h after I/R injury (*n* = 5). (H) Representative M‐mode echocardiographic images acquired 3 weeks after I/R injury (*n* = 5). (I) Quantitative analysis of echocardiographic parameters. EF, ejection fraction; FS, fractional shortening; LVIDd, left ventricular internal diameter at end‐diastolic phase; LVIDs, left ventricular internal diameter at end‐systolic phase (*n* = 5). (J) H&E staining and Masson's trichrome staining of cardiac sections at 3 weeks after I/R injury (*n* = 5). Scale bar: 100 or 1000 µm. (K) Scar size quantified as the percentage of the left ventricular area (*n* = 5). (L) H&E staining of liver, kidney, and lung sections from vehicle‐ and STM2457‐treated mice at 3 weeks after I/R injury (*n* = 5). Scale bar: 100 or 1000 µm. All data are presented as mean ± SD. ns indicates not significant; *p < 0.05; **p < 0.01; ***p < 0.001; ****p < 0.0001.

We next evaluated the protective effect of pharmacological METTL3 inhibition in vivo. C57BL/6J mice were intraperitoneally administered STM2457 (30 mg/kg) every 12 h, beginning 1 day before I/R surgery and continuing through postoperative day 5 (Figure 8A). Compared with vehicle treatment, STM2457 reduced multiple PANoptosis‐related death signaling proteins in myocardial tissue during the early phase of I/R injury (Figure 8B–E). STM2457 also reduced myocardial infarct size at 1 day after I/R injury, as assessed by Evans blue/TTC staining and quantification of the area at risk and infarct area (Figure 8F,G). At 3 weeks, echocardiography showed improved cardiac function, with increased EF and FS and reduced LVIDd and LVIDs (Figure 8H,I). Histological analyses further demonstrated reduced myocardial fibrosis and scar formation (Figure 8J,K). No overt histopathological abnormalities were observed in the liver, lungs, or kidneys under this treatment regimen (Figure 8L).

Together, these findings indicate that STM2457 treatment attenuates early PANoptosis‐related death signaling, reduces acute myocardial injury, and alleviates subsequent cardiac dysfunction and adverse remodeling after I/R injury, supporting METTL3 inhibition as a potential protective strategy in this experimental setting.

## 3 Discussion

In this study, METTL3 was upregulated in I/R‐injured myocardium and OGD/R‐treated cardiomyocytes, partly through p300‐dependent H3K18la. Mechanistically, METTL3 enhanced m^6^A modification of *Egr1* mRNA and promoted EGR1 expression through YTHDF1‐associated translational regulation and IGF2BP2‐mediated mRNA stabilization. Elevated EGR1 activated *Zbp1* transcription and facilitated interactions between ZBP1 and PANoptosis‐related components, thereby contributing to PANoptosis‐like cardiomyocyte death. Genetic rescue experiments confirmed that EGR1 mediated the detrimental effects of METTL3, while STM2457 reduced infarct size, improved cardiac function, and attenuated fibrosis. Collectively, these findings delineate a novel epigenetic‐epitranscriptomic axis involving H3K18la, METTL3, EGR1, and ZBP1 that promotes PANoptosis‐like cardiomyocyte death during myocardial I/R injury.

Previous studies have shown that p300‐mediated H3K27 acetylation activates METTL3 transcription in gastric cancer [57], and that c‐Jun induces METTL3 expression by binding to its promoter [58], suggesting that METTL3 expression is governed by multilayered regulation involving multiple signaling pathways. Lactate is increasingly recognized as a metabolic signal that drives lysine lactylation and influences gene regulation in cancer and ischemic heart disease [59, 60, 61, 62, 63], while histone and non‐histone lactylation contribute to broader disease processes [64, 65, 66, 67]. In our models, lactate accumulation coincided with increased H3K18la and METTL3 expression. Moreover, p300 knockdown suppressed METTL3 induction, while pharmacological p300 inhibition reduced both H3K18la and METTL3 levels. Increased H3K18la enrichment at the *Mettl3* promoter and enhanced promoter activity further supported a link between histone lactylation and *Mettl3* transcription. However, I/R injury activates interconnected metabolic, inflammatory, oxidative, and chromatin‐regulatory pathways. Thus, lactate/H3K18la‐associated METTL3 upregulation is unlikely to be the sole upstream mechanism. Rather, our findings identify H3K18la as an experimentally supported link between metabolic remodeling and *Mettl3* transcriptional regulation that likely acts alongside other ischemia‐associated signals and chromatin regulators. Notably, lactate and lactylation may exert context‐dependent effects during I/R injury. A recent study reported that K351 lactylation of Serpina3k/SA3K enhanced its protein stability and enabled fibroblast‐derived lactylated SA3K to protect cardiomyocytes from I/R‐induced apoptosis [68]. These findings indicate that lactate‐driven lactylation can have either detrimental or protective consequences depending on the modified target, cellular source, and temporal context.

PANoptosis integrates pyroptotic, apoptotic, and necroptotic signaling and can amplify inflammatory tissue injury [51]. Although PANoptosis has been extensively studied in immune and cancer contexts [28, 69], its role in the cardiovascular system remains poorly understood. In the cardiovascular system, I/R injury has been linked to the independent activation of pyroptosis [70], apoptosis [71], and necroptosis [72] in cardiomyocytes; however, whether these death programs are coordinated by a shared upstream mechanism remains unclear. In this study, we observed a concomitant increase in representative markers of pyroptosis, apoptosis, and necroptosis during early reoxygenation or reperfusion, which temporally paralleled METTL3 upregulation. Genetic manipulation of METTL3 expression and pharmacological inhibition of its enzymatic activity consistently modulated these death signals and associated pathological outcomes, including cardiomyocyte injury, infarct size, cardiac dysfunction, and fibrosis, supporting METTL3 as an upstream regulator of PANoptosis‐related signaling during myocardial I/R injury.

PANoptosome assembly is a defining feature of PANoptosis [30]. ZBP1, an innate immune sensor of Z‐RNA/Z‐DNA, is a major PANoptosome trigger, particularly during viral infection [30, 34, 73], where it can engage RIPK3, RIPK1, Caspase‐8, ASC, and NLRP3 to coordinate cell‐death signaling [30, 73]. In OGD/R‐treated cardiomyocytes, ZBP1 was strongly induced and responded to METTL3 modulation. ZBP1 knockdown attenuated METTL3‐driven pyroptotic, apoptotic, and necroptotic signaling. Enhanced associations and spatial co‐localization of ZBP1 with RIPK3, Caspase‐8, and Caspase‐6 were observed in OGD/R‐treated cardiomyocytes and I/R‐injured myocardium, whereas ZBP1 depletion weakened these interactions and reduced downstream death signals. These findings support a ZBP1‐associated PANoptosis‐related signaling module in cardiomyocytes. Importantly, this module may not fully recapitulate canonical infection‐associated PANoptosomes, and its molecular composition is likely shaped by the sterile injury environment and cellular context of myocardial I/R injury.

EGR1 is a stress‐responsive zinc‐finger transcription factor involved in proliferation, differentiation, apoptosis, and inflammation [74, 75, 76]. Its inhibition attenuates hypertrophic remodeling [77], while EGR1 deficiency reduces inflammatory and procoagulant signaling and slows atherosclerosis [78, 79]. EGR1 has also been implicated in I/R‐induced cardiomyocyte apoptosis [80, 81], but its role in PANoptosis‐related cardiomyocyte death remained unclear. Our findings identify EGR1 as a functional link between METTL3‐dependent m^6^A regulation and ZBP1‐associated PANoptosis‐related signaling. METTL3 promoted EGR1 expression, whereas EGR1 enhanced *Zbp1* transcription; accordingly, EGR1 silencing was protective, while EGR1 restoration partly reversed the effects of METTL3 deficiency.

The fate of m^6^A‐modified transcripts depends on reader proteins, particularly the YTH and IGF2BP families [82]. YTH proteins regulate splicing, decay, and translation [83], and YTHDF1 can promote the translation of m^6^A‐modified transcripts such as Braf [84]. IGF2BP proteins mainly stabilize target RNAs [85]; for example, IGF2BP2 stabilizes m^6^A‐modified *TAB3* mRNA during acute kidney injury [86]. Here, YTHDF1 and IGF2BP2 regulated EGR1 through complementary mechanisms: YTHDF1 mainly promoted *Egr1* translation, whereas IGF2BP2 prolonged *Egr1* mRNA stability. Although METTL3‐dependent changes in the association between eEF1A1 and *Egr1* mRNA were observed, a stable direct YTHDF1‐eEF1A1 interaction was not established. This finding should therefore be considered exploratory rather than evidence of a complete recruitment mechanism.

Therapeutic targeting of METTL3 remains at an early stage, but preclinical studies suggest potential in acute inflammation and tissue injury [87]. STM2457 suppresses acute myeloid leukemia [47] and several solid tumors [88, 89, 90] and has shown protective effects in renal fibrosis and doxorubicin‐induced cardiomyopathy [37, 91]. In our study, STM2457 suppressed PANoptosis‐related signaling, reduced infarct size, preserved cardiac function, and limited fibrosis. Because treatment continued through reperfusion and the post‐reperfusion phase, these data support METTL3 inhibition as a strategy to restrain early cardiomyocyte death and subsequent remodeling. However, the pretreatment period limits direct extrapolation to clinical post‐ischemic therapy.

Nevertheless, several limitations should be acknowledged. First, although our findings support PANoptosis‐related death signaling in myocardial I/R injury, definitive evidence of PANoptosome‐driven integrated cell death at single‐cell resolution remains lacking. Proximity‐based assays, dynamic imaging, single‐cell analyses, and conditional disruption of key components are needed to further validate PANoptosome formation and function. Second, given the pleiotropic effects of lactate, locus‐specific chromatin approaches are required to confirm the directness and specificity of H3K18la‐mediated METTL3 regulation. Third, the endogenous nucleic acid ligands that activate ZBP1 during sterile myocardial I/R injury remain unknown. Finally, because STM2457 was administered before ischemia, future studies should define clinically relevant reperfusion‐onset and post‐reperfusion treatment windows and evaluate its pharmacokinetics, pharmacodynamics, and long‐term safety.

## 4 Materials and Methods

### 4.1 Cell Culture and Treatments

HL‐1 (RRID: CVCL_0303; catalog no. ZQ0920) and AC16 (RRID: CVCL_4U18; catalog no. ZQ0960) cell lines were purchased from Shanghai Zhong Qiao Xin Zhou Biotechnology Co., Ltd. (Shanghai, China). Cell identities were authenticated by supplier‐performed short tandem repeat profiling, and all cultures were routinely confirmed to be mycoplasma‐free. HL‐1 and AC16 cells were maintained in high‐glucose DMEM supplemented with 10% FBS and penicillin/streptomycin at 37°C with 5% CO_2_. NMCMs were isolated from 1‐ to 2‐day‐old C57BL/6J mice as previously described and cultured in DMEM/F12 containing 10% FBS and penicillin/streptomycin. OGD/R was induced by culturing cells in serum‐ and glucose‐free DMEM under 95% N_2/_5% CO_2_, followed by restoration of normoxia and high‐glucose DMEM containing 10% FBS. Unless otherwise indicated, OGD and reoxygenation were each performed for 3 h. For inhibitor experiments, cells were pretreated with STM2457 (MCE, USA; HY‐134836) for 24 h before OGD/R.

### 4.2 Cell Transfection, Plasmids, and siRNAs

Lentiviral vectors and gene‐specific siRNAs were obtained from Hanheng Biotechnology (Shanghai, China). For transient overexpression, the coding sequences of METTL3 and EGR1 were separately cloned into pcDNA3.1. Stable METTL3‐knockdown cells were generated using a lentiviral METTL3‐targeting shRNA followed by puromycin selection. Plasmids and siRNAs were transfected using Lipofectamine 3000 and P3000 reagents according to the manufacturer's instructions. siRNA sequences are listed in Table S5.

### 4.3 Animals and Experimental Procedures

Neonatal C57BL/6J mice were purchased from the Shanghai Laboratory Animal Center (Shanghai, China). Male C57BL/6J mice (6–8 weeks old, 22–26 g), *METTL3^fl/fl^* mice, and *Myh6‐CreERT2* transgenic mice were obtained from Gempharmatech (Nanjing, China). Tamoxifen‐inducible cardiomyocyte‐specific *Mettl3* knockout mice (*Mettl3^fl/fl^; Myh6‐CreERT2^+^*, METTL3‐CKO) were generated by crossing *Mettl3^fl/fl^* mice with *Myh6‐CreERT2* mice, with *Mettl3^fl/fl^; Myh6‐CreERT2^−^* littermates serving as controls (METTL3‐WT). Both genotypes received intraperitoneal injections of tamoxifen (100 mg/kg/day; Sigma‐Aldrich, USA; T5648) for five consecutive days, and subsequent experiments were performed 2 weeks after the final injection. For pharmacological inhibition of METTL3, wild‐type mice received intraperitoneal injections of STM2457 (30 mg/kg; Selleck Chemicals, China; S9870) or vehicle containing 10% DMSO and 90% saline every 12 h, beginning 24 h before I/R surgery and continuing through postoperative Day 5.

All animals were housed under specific pathogen‐free conditions on a 12‐h light/dark cycle at 22 ± 2°C and 50%–60% relative humidity, with ad libitum access to food and water. Animals were randomly assigned to the experimental groups using a computer‐generated randomization sequence. Group sizes of four to five animals were selected based on preliminary experiments and sample sizes commonly used in previous murine myocardial I/R studies, with consideration of the expected effect size, biological variability, animal welfare, and experimental feasibility. No formal a priori power calculation was performed. Only healthy animals exhibiting normal appearance, activity, and body weight were enrolled. Mice with congenital abnormalities or evidence of illness were excluded before enrollment. Animals that died before the prespecified endpoint were excluded from the corresponding analyses and reported by group. No other animals or data points were excluded. Investigators performing animal allocation and experimental procedures were aware of group assignment. Echocardiographic, histological, and image analyses were performed using coded samples by investigators blinded to group allocation. For all in vivo experiments, *n* represents the number of individual animals. The study was designed and reported in accordance with the ARRIVE 2.0 guidelines [92].

### 4.4 Myocardial Ischemia‐Reperfusion Model

To establish cardiomyocyte‐specific gene manipulation models, male C57BL/6J mice received tail‐vein injections of HBAAV2/9‐cTNT vectors encoding *Mettl3*, *Egr1*, a *Zbp1*‐targeting shRNA, or the corresponding controls at a dose of 2 × 10^11^ vector genomes per mouse in a total volume of 100 µL (Hanheng Biotechnology, Shanghai, China). Three weeks after viral administration, mice were anesthetized with 2.0% isoflurane, endotracheally intubated, and mechanically ventilated. Myocardial ischemia was induced by ligation of the left anterior descending coronary artery with a 6‐0 silk suture for 45 min, followed by reperfusion. Successful ischemia and reperfusion were confirmed by changes in myocardial color. Sham‐operated mice underwent the same surgical procedure without coronary artery ligation. Separate cohorts of animals were used for acute molecular analyses at 24 h, infarct‐size assessment at 24 h, and cardiac functional and histological assessments at 3 weeks after reperfusion. At the designated endpoints, mice were euthanized by intraperitoneal administration of pentobarbital sodium (200 mg/kg), and death was confirmed by the absence of cardiac activity and reflex responses. For acute analyses, viable tissue from the peri‐infarct border zone was used for biochemical assays, while heart sections were used for immunostaining. In the long‐term cohort, transthoracic echocardiography was performed 3 weeks after I/R injury, followed by hematoxylin and eosin (H&E) and Masson's trichrome staining to evaluate myocardial architecture and fibrosis.

### 4.5 Measurement of Lactate Levels

After completing the OGD/R treatment, intracellular lactate levels in NMCMs and HL‐1 cells were measured using a colorimetric assay kit (Solarbio, China; BC2235) according to the manufacturer's instructions.

### 4.6 Western Blot Analysis

Proteins were extracted from cells and tissues using RIPA lysis buffer (Beyotime Biotechnology, China; P0013B) supplemented with protease and phosphatase inhibitors (Beyotime Biotechnology, China; P1045). Protein concentrations were determined using the Micro BCA Protein Assay Kit (Beyotime Biotechnology, China; P0012). Equal amounts of protein were separated by SDS‐PAGE and transferred to PVDF membranes (Millipore, USA; IPVH00010). Membranes were blocked with 5% BSA, incubated with primary antibodies overnight at 4°C, and then with HRP‐conjugated secondary antibodies for 1 h at room temperature. Protein bands were visualized using a chemiluminescence imaging system. Antibodies are listed in Table S1.

### 4.7 Quantitative Real‐Time PCR (qPCR) Analysis

Total RNA was extracted using the UNlQ‐10 Column Total RNA Purification Kit (Sangon Biotech, China; B511361) and then reverse transcribed into cDNA using Hifair II first Strand cDNA Synthesis Kit (Yeasen, China; 11119ES60). Quantitative real‐time PCR (qPCR) analysis was conducted using Hifair qPCR SYBR Green Master Mix (Yeasen, China; 11201ES08) on an ABI QuantStudio5 (Thermo Fisher Scientific, Waltham, USA). Relative mRNA expression levels were calculated using the 2^−ΔΔ^ *^Ct^* method and normalized to β‐actin. The sequences of primers used are presented in Table S2.

### 4.8 Cell Viability Assay

Cell viability was assessed using the Cell Counting Kit‐8 (CCK‐8; Beyotime Biotechnology, China; C0038). HL‐1 or AC16 cells were seeded in 96‐well plates at 5000 cells per well. After treatment, cells were incubated with 10 µL of CCK‐8 reagent for 1.5 h at 37°C, and absorbance at 450 nm was measured using a microplate reader (Bio‐Tek, USA).

### 4.9 Propidium Iodide (PI), TUNEL, and Ethidium Homodimer III (EthD‐III) Staining

Cell death was assessed using PI/Calcein‐AM staining (Beyotime Biotechnology, China; C2015S), a TUNEL apoptosis detection kit (Beyotime Biotechnology, China; C1089), and EthD‐III staining (Biotium, USA; 40050) according to the manufacturers’ instructions. Briefly, treated HL‐1 or AC16 cells were stained with PI/Calcein‐AM for 30 min at 37°C, subjected to TUNEL staining for 60 min at 37°C after fixation and permeabilization, or incubated with EthD‐III for 10 min at room temperature. TUNEL‐ and EthD‐III‐stained cells were counterstained with DAPI. Images from three random fields per well were acquired by fluorescence microscopy, and the percentage of positively stained cells was quantified using ImageJ.

### 4.10 Immunofluorescence and Immunohistochemistry

For cellular immunofluorescence, HL‐1 cells were fixed with 4% paraformaldehyde, permeabilized with 0.2% Triton X‐100, blocked with 10% goat serum, and incubated with primary antibodies overnight at 4°C. Cells were then stained with fluorophore‐conjugated secondary antibodies and DAPI.

For tissue immunofluorescence and immunohistochemistry, paraffin‐embedded tissues were sectioned at 4 µm, deparaffinized, rehydrated, and subjected to heat‐induced antigen retrieval in Tris‐EDTA buffer (pH 9.0). For immunofluorescence, sections were blocked with 5% BSA and incubated with primary antibodies for 2 h at room temperature, followed by fluorophore‐conjugated secondary antibodies and DAPI staining. For immunohistochemistry, endogenous peroxidase activity was blocked with 3% hydrogen peroxide, and sections were incubated with primary antibodies overnight at 4°C, followed by HRP‐conjugated secondary antibodies and DAB development. Images were acquired by fluorescence or light microscopy and analyzed using ImageJ. Antibodies are listed in Table S4.

### 4.11 Evans Blue/TTC Double Staining

At 24 h after I/R injury, the LAD was re‐occluded at the original ligation site, and 1 mL of 1% Evans blue dye (Nanjing Senbeijia Biotechnology, China; BP‐DL502) was injected into the left ventricle to delineate the non‐ischemic myocardium. Hearts were harvested and cut into 1‐mm‐thick transverse slices, which were incubated with 2% TTC (Merck, Germany; 108380) for 30 min. The area at risk relative to the left ventricle (AAR/LV) and infarct size relative to the area at risk (INF/AAR) were quantified.

### 4.12 Luciferase Reporter Assays

To evaluate EGR1‐mediated *Zbp1* transcription, the *Zbp1* promoter region (−600 to +100 bp) was cloned into pGL3‐Basic, and the predicted EGR1‐binding site (−279 to −266 bp) was mutated. HL‐1 cells were co‐transfected with the wild‐type or mutant reporter and the TK‐Renilla control for 36 h. A pGL3‐Basic‐Mettl3 promoter construct was used to assess p300‐dependent changes in *Mettl3* promoter activity under OGD/R conditions.

For m^6^A‐site validation, the *Egr1* coding sequence and 3′ untranslated region were separately cloned into the pmirGLO vector. Individual A‐to‐T substitutions were introduced at four candidate sites in the CDS and one site in the 3′UTR. Luciferase activity was measured using the Dual‐Luciferase Reporter Assay Kit (Beyotime Biotechnology, China; RG005) and normalized to Renilla luciferase activity.

### 4.13 Extraction of Cytoplasmic and Nuclear Proteins

Cytoplasmic and nuclear proteins were extracted using the Nuclear and Cytoplasmic Protein Extraction Kit (Beyotime Biotechnology, China; P0027) according to the manufacturer's instructions.

### 4.14 Quantification of Global RNA m^6^A Levels

Global m^6^A levels in total RNA were measured using the m^6^A RNA Methylation Quantification Kit (Epigentek, USA; P‐9005‐96) according to the manufacturer's instructions. A total of 200 ng RNA was used per well, and m^6^A content was calculated from a standard curve generated using the kit‐provided positive control.

### 4.15 MeRIP‐seq

Total RNA was extracted using TRIzol reagent (Thermo Fisher Scientific, USA; 15596018), treated with DNase I (Roche Diagnostics, Switzerland; 04716728001), and fragmented in a buffer containing 100 mM Tris‐HCl and 100 mM ZnCl_2_. Fragmented RNA was divided into input and immunoprecipitation fractions. Protein A/G magnetic beads (Thermo Fisher Scientific, USA; 10002D and 10004D) were conjugated with 5 µg of anti‐m^6^A antibody (Abcam, USA; ab151230) for at least 6 h at 4°C and then incubated with fragmented RNA for 2 h. After sequential washing, m^6^A‐enriched RNA was purified using the RNeasy Mini Kit (QIAGEN, Germany; 74106). Strand‐specific libraries were prepared using the SMARTer Stranded Total RNA‐Seq Kit v2 (Takara Bio, Japan; 634413) and subjected to paired‐end sequencing on an Illumina NovaSeq platform.

Raw reads were processed using Trimmomatic [93] and aligned to the mouse reference genome using STAR [94]. m^6^A‐enriched peaks were identified using MetPeak [95] and annotated using ChIPseeker [96]. Gene expression in input libraries was quantified as fragments per kilobase of transcript per million mapped reads (FPKM) using StringTie [97]. Differentially methylated peaks were identified using MeTDiff with thresholds of |log_2_(fold change)| > 1 and *p* value < 0.05 [98].

### 4.16 Co‐Immunoprecipitation (Co‐IP), Chromatin Immunoprecipitation‐qPCR (ChIP‐qPCR), and RNA Immunoprecipitation‐qPCR (RIP‐qPCR) Analyses

Co‐IP, ChIP, and RIP assays were performed using the corresponding kits from GENESEED (China; P0401, P0301, and P0101, respectively) according to the manufacturer's instructions.

For Co‐IP, whole‐cell lysates prepared in NP‐40 buffer containing protease and phosphatase inhibitors were incubated overnight at 4°C with antibody‐conjugated protein A/G magnetic beads. Antibodies against METTL3 (Abcam, ab195352), ZBP1 (Adipogen, AG‐20B‐0010‐C100), RIPK3 (ProSci, 2283), or control IgG (CST, 5127) were used. Immunoprecipitated proteins were analyzed by Western blotting.

For ChIP‐qPCR, cells were cross‐linked with 1% formaldehyde, and chromatin was sheared into 200–300 bp fragments. Chromatin was immunoprecipitated overnight at 4°C using antibodies against H3K18la (PTM BIO, PTM‐1406RM), EGR1 (CST, 4154T), or control IgG (CST, 5127). Purified DNA was analyzed by qPCR using Hifair qPCR SYBR Green Master Mix (Yeasen, China; 11201ES08). ChIP‐qPCR primer sequences are listed in Table S3.

For RIP‐qPCR, a portion of each cell lysate was retained as the input, while the remaining lysate was incubated with antibodies against METTL3 (Abcam, ab195352), YTHDF1 (Abcam, ab220162), IGF2BP2 (Proteintech, 11601‐1‐AP), eEF1A1 (ABclonal, A23515), eEF2 (ABclonal, A9721), or control IgG (CST, 5127). Immunoprecipitated and input RNA were purified and analyzed by RT‐qPCR.

### 4.17 Protein and mRNA Stability Analysis

For mRNA stability analysis, cells were transfected with control or target‐gene siRNA for 36 h and then treated with actinomycin D (5 µM; MCE, China; HY‐17559). Cells were collected at the indicated time points, and mRNA levels were measured by RT‐qPCR. EGR1 protein stability was assessed by Western blotting after treatment with cycloheximide (100 µM; MCE, China; HY‐12320).

### 4.18 Polysome Profiling

Control and METTL3‐knockdown cells were treated with cycloheximide (100 µM; MCE, China; HY‐12320) for 10 min at 37°C and lysed on ice. Clarified lysates were layered onto 5%–50% sucrose density gradients and ultracentrifuged at 36,000 rpm for 3 h at 4°C using an Optima XE‐90 ultracentrifuge (Beckman Coulter, Germany). The gradients were fractionated using a UV‐monitored Gradient Station (BioComp Instruments, Canada). RNA was extracted from the collected fractions, and the abundance of *Egr1* mRNA in each fraction was determined by RT‐qPCR.

### 4.19 SELECT for Determination of m^6^A Sites

A SELECT assay was performed to quantify site‐specific m^6^A modification at the A1846 and A1921 sites of *Egr1* mRNA according to the manufacturer's instructions.

### 4.20 Statistical Analysis

Statistical analyses were performed using GraphPad Prism 9.0 (GraphPad Software, La Jolla, CA, USA). Data are presented as the mean ± standard deviation (SD). Comparisons between two independent groups were performed using a two‐tailed unpaired Student's *t*‐test. For comparisons among multiple groups, one‐way ANOVA was performed, followed by Bonferroni's test for predefined pairwise comparisons or Dunnett's test when multiple experimental groups were compared with a single control. For experiments involving two independent variables, two‐way ANOVA followed by Bonferroni's post hoc test was used where appropriate. If significant main effects or interactions were detected, Bonferroni correction was applied for predefined post hoc comparisons. The number of biological replicates or independent experiments (n) is indicated in the corresponding figure legends. A value of *p* < 0.05 was considered statistically significant. Statistical annotations in the figures are defined as follows: ns, not significant; *p < 0.05, **p < 0.01, ***p < 0.001, and ****p < 0.0001.

## Author Contributions

L.L. and H.C. contributed to the conception and design of the study. L.L. and J.G. performed the cell and animal experiments. X.Z. performed the data analysis. K.D. supervised the study. J.G. and J.M. provided experimental resources. L.L. drafted the manuscript. J.M. reviewed and revised the manuscript. All authors have read and approved the final manuscript.

## Funding

This work was financially supported by the National Natural Science Foundation of China (T2288101).

## Ethics Statement

All animal procedures were conducted in accordance with the National Institutes of Health Guide for the Care and Use of Laboratory Animals and were approved by the Animal Care and Use Committee of Zhongshan Hospital, Fudan University (Approval No. 2025–096).

## Conflicts of Interest

All authors declare no conflicts of interest.

## Supporting information




**Supporting File**: mco270962‐sup‐0001‐SuppMat.docx.

## Data Availability

The MeRIP‐seq raw sequencing data generated in this study have been deposited in the NCBI Sequence Read Archive under BioProject accession number PRJNA1472439 and SRA accession number SRP704767, and will be publicly available upon publication. All other data supporting the findings of this study are available within the article and its Supporting Information, or from the corresponding author upon reasonable request.
