# Supplementary material for: METTL3‐Mediated N6‐Methyladenosine Modification Activates the EGR1‐ZBP1 Axis to Promote PANoptosis‐Like Cardiomyocyte Death in Myocardial Ischemia‐Reperfusion Injury
Source: MedComm (2020). 2026 Sep 21;7(9):e70962. doi: 10.1002/mco2.70962 (PMC13591228; doi:10.1002/mco2.70962)
Supplement: Supplementary file 1 [file MCO2-7-e70962-s001.docx]

**METTL3-mediated N6-methyladenosine modification activates the EGR1-ZBP1 axis to promote PANoptosis-like cardiomyocyte death in myocardial ischemia-reperfusion injury**

Linnan Li, MD, PHD^1, 2, #^, Jinfeng Gao, MD, PHD^1, 2, #^, Xiaoxue Zhang, MD, PHD^1, 2, #^, Kunming Dai, MD, PHD^1, 2^, Hao Cheng, MD, PHD^1, 2^, Jianying Ma, MD, PHD^1, 2, *^, Junbo Ge, MD, PHD^1, 2, 3, 4, 5, 6, *^

1.Department of Cardiology, Zhongshan Hospital, Fudan University, Shanghai Institute of Cardiovascular Diseases, China.

2.State Key Laboratory of Cardiovascular Diseases, Zhongshan Hospital, Fudan University.
3.NHC Key Laboratory of Ischemic Heart Diseases.
4.Key Laboratory of Viral Heart Diseases, Chinese Academy of Medical Sciences.
5.National Clinical Research Center for Interventional Medicine, Shanghai, China.
6.Institutes of Biomedical Sciences, Fudan University, Shanghai, China.

* Correspondence: Jianying Ma ([ma.jianying@zs-hospital.sh.cn](mailto:ma.jianying@zs-hospital.sh.cn)), Junbo Ge ([jbge@zs-hospital.sh.cn](mailto:jbge@zs-hospital.sh.cn)).

\# These authors contributed equally to this work.

**SUPPLEMENTARY FIGURES AND FIGURE LEGENDS**

**Figure S1. OGD/R induces METTL3 upregulation in HL-1 cells.**

(A, B) Western blot analysis and quantification of METTL3 protein levels in HL-1 cells exposed to OGD for 0, 3, 6, or 12 h, followed by 12 h of reoxygenation (n = 3). (C, D) Western blot analysis of METTL3 protein levels in HL-1 cells subjected to OGD for 3 h, followed by 1, 3, 6, 12, or 24 h of reoxygenation (n = 3). All data are presented as mean ± SD. ns indicates not significant; **P* < 0.05; ***P* < 0.01; ****P* < 0.001; *****P* < 0.0001.

**Figure S2. Lactate-associated p300-dependent H3K18la contributes to METTL3 transcriptional upregulation in OGD/R-treated HL-1 cells.**

(A) L-lactic acid concentrations in HL-1 cells from the control and OGD/R groups were measured using a lactic acid assay kit (n = 4). (B, C) Western blot analysis and quantification of METTL3 protein levels in HL-1 cells transfected with siRNA targeting LDHA for 48 h under OGD/R conditions (n = 3). (D, E) Western blot analysis and quantification of METTL3 protein expression in HL-1 cells pretreated with 20 mM 2-DG or 500 nM rotenone for 24 h under OGD/R conditions (n = 3). (F, G) Western blot analysis of METTL3 protein expression in HL-1 cells treated with 25 mM L-lactic acid for various time periods (n = 3). (H, I) Western blot analysis of the indicated proteins in HL-1 cells following OGD/R treatment (n = 3). (J) RT-qPCR analysis of *Ep300* and *Mettl3* mRNA levels in control and p300-knockdown HL-1 cells under OGD/R conditions (n = 3). (K, L) Western blot analysis of the indicated proteins in control and p300-knockdown HL-1 cells under control or OGD/R conditions (n = 3). (M, N) Western blot analysis of the indicated proteins in HL-1 cells pretreated with or without 100 μM C646 for 24 h under control or OGD/R conditions (n = 3). (O) IP with anti-METTL3 followed by pan-Kla immunoblotting to assess whether METTL3 underwent lactylation under OGD/R conditions (n = 3). All data are presented as mean ± SD. ns indicates not significant; **P* < 0.05; ***P* < 0.01; ****P* < 0.001; *****P* < 0.0001.

**Figure S3. METTL3 promotes PANoptosis-like cell death in OGD/R-treated AC16 cells.**

(A, B) Representative images of propidium iodide (PI) staining in AC16 cells following the indicated treatments (n = 5). Scale bar: 100 μm. (C, D) Representative images of EthD-III staining in AC16 cells following the indicated treatments (n = 5). Scale bar: 100 μm. (E, F) Representative images of TUNEL staining in AC16 cells following the indicated treatments (n = 5). Scale bar: 100 μm. All data are presented as mean ± SD. ns indicates not significant; **P* < 0.05; ***P* < 0.01; ****P* < 0.001; *****P* < 0.0001.

**Figure S4. Pharmacological inhibition reveals crosstalk among apoptosis-, necroptosis-, and pyroptosis-related pathways in OGD/R-treated NMCMs.**

(A, B) Western blot analysis of the indicated proteins in NMCMs pretreated with 10 μM Q-VD-OPh for 2 h, followed by OGD/R in the continued presence of Q-VD-OPh (n = 3). (C, D) Western blot analysis of the indicated proteins in NMCMs pretreated with 3 μM GSK-872 for 2 h, followed by OGD/R in the continued presence of GSK-872 (n = 3). (E, F) Western blot analysis of the indicated proteins in NMCMs pretreated with 10 μM MCC950 for 2 h, followed by OGD/R in the continued presence of MCC950 (n = 3). All data are presented as mean ± SD. ns indicates not significant; **P* < 0.05; ***P* < 0.01; ****P* < 0.001; *****P* < 0.0001.

**Figure S5. Cardiomyocyte-specific METTL3 overexpression exacerbates I/R-induced PANoptosis-related death signaling and aggravates cardiac dysfunction and adverse remodeling.**

(A, B) Western blot analysis of the indicated proteins in myocardial tissue from OE-Control and OE-METTL3 mice at 1 day post-I/R injury (n = 3). (C, D) Immunohistochemical staining of cardiac sections from OE-Control and OE-METTL3 mice at 1 day post-I/R injury (n = 5). Scale bar: 100 μm. (E, F) Representative images of TTC-stained transverse sections of hearts after Evans blue perfusion. The AAR and infarct size were measured 24 h after I/R injury (n = 5). (G) Representative M-mode echocardiography images at 3 weeks post-I/R injury (n = 4-5). (H) Quantitative analysis of echocardiographic parameters. EF, ejection fraction; FS, fractional shortening; LVIDd, left ventricular internal diameter at end-diastolic phase; LVIDs, left ventricular internal diameter at end-systolic phase (n = 4-5). (I) H&E staining and Masson’s Trichrome staining of cardiac sections at 3 weeks post-I/R injury (n = 4-5). Scale bar: 100 μm or 1000 μm. (J) Scar size quantified as the percentage of the left ventricular area (n = 4-5). All data are presented as mean ± SD. ns indicates not significant; **P* < 0.05; ***P* < 0.01; ****P* < 0.001; *****P* < 0.0001.

**Figure S6. METTL3 regulates EGR1 expression in OGD/R-treated AC16 and HL-1 cells.**

(A, B) mRNA expression levels of *METTL3* and candidate genes in control and METTL3-overexpressing (A) or METTL3-knockdown (B) AC16 cells under OGD/R conditions (n = 3). (C-N) Western blot analysis of EGR1 and ATF3 protein levels in AC16 cells following METTL3 overexpression (C, D), METTL3 knockdown (E, F), or STM2457 treatment (G, H); and in HL-1 cells under the same respective conditions (I, J; K, L; M, N) under OGD/R conditions (n = 3). All data are presented as mean ± SD. ns indicates not significant; **P* < 0.05; ***P* < 0.01; ****P* < 0.001; *****P* < 0.0001.

**Figure S7. EGR1 mediates METTL3-driven PANoptosis-related death signaling and cell death phenotypes in OGD/R-treated cardiomyocytes.**

(A, B) Western blot analysis of the indicated proteins in control and EGR1-overexpressing (A) or EGR1-knockdown (B) NMCMs under control or OGD/R conditions (n = 3). (C) NMCMs were transduced with lentivirus to overexpress EGR1 for 24 h, followed by siRNA-mediated knockdown of ZBP1 for an additional 24 h before exposure to OGD/R. Western blot analysis was performed to assess the indicated proteins (n = 3). (D) NMCMs were transduced with lentiviral shRNA to knock down METTL3 for 24 h, followed by lentiviral transduction to overexpress EGR1 for an additional 24 h under control or OGD/R conditions. Western blot analysis was performed for the indicated proteins (n = 3). (E) Cell viability was assessed using the CCK-8 assay (n = 4). (F) NMCMs were transduced with lentivirus to overexpress METTL3 for 24 h, followed by siRNA-mediated knockdown of EGR1 for an additional 24 h under either control or OGD/R conditions. Western blot analysis was performed to assess the indicated proteins (n = 3). (G) Cell viability was assessed using the CCK-8 assay (n = 4). (H, I) Representative images of PI staining in HL-1 cells under the indicated conditions (n = 5). Scale bar: 100 μm. (J, K) Representative images of EthD-III staining in HL-1 cells under the indicated conditions (n = 5). Scale bar: 100 μm. (L, M) Representative images of TUNEL staining in HL-1 cells under the indicated conditions (n = 5). Scale bar: 100 μm. All data are presented as mean ± SD. ns indicates not significant; **P* < 0.05; ***P* < 0.01; ****P* < 0.001; *****P* < 0.0001.

**Figure S8. EGR1 overexpression partially reverses the protective effects of cardiomyocyte-specific METTL3 knockout against I/R injury.**

(A, B) Western blot analysis of the indicated proteins in myocardial tissue from control and EGR1-overexpressing METTL3-CKO mice at 1 day after I/R injury (n = 3). (C, D) Immunohistochemical staining of cardiac sections from control and EGR1-overexpressing METTL3-CKO mice at 1 day after I/R injury (n = 5). Scale bar: 100 μm. (E, F) Representative images of TTC-stained transverse sections of hearts after Evans blue perfusion. The AAR and infarct size were measured 24 h after I/R injury (n = 5). (G) Representative M-mode echocardiography images obtained at 3 weeks after I/R injury (n = 5). (H) Quantitative analysis of echocardiographic parameters. EF, ejection fraction; FS, fractional shortening; LVIDd, left ventricular internal diameter at end-diastolic phase; LVIDs, left ventricular internal diameter at end-systolic phase (n = 5). (I) H&E staining and Masson’s Trichrome staining of cardiac sections at 3 weeks after I/R injury (n = 5). Scale bar: 100 μm or 1000 μm. (J) Scar size quantified as the percentage of the left ventricular area (n = 5). All data are presented as mean ± SD. ns indicates not significant; **P* < 0.05; ***P* < 0.01; ****P* < 0.001; *****P* < 0.0001.

**Figure S9. METTL3 enhances *Egr1* mRNA stability and translational efficiency via m^6^A modification.**

(A) MeRIP-seq analysis showing the distribution and abundance of m^6^A modifications on *Egr1* mRNA. (B) Predicted m^6^A modification sites in mouse *Egr1* mRNA based on MeRIP-seq-identified differential peaks using the SRAMP algorithm. (C, D) RIP-qPCR analysis of the interaction between METTL3 and *Egr1* mRNA in HL-1 cells under OGD/R conditions (C) or following METTL3 overexpression (D). Enrichment of *Egr1* mRNA was normalized to input (n = 4). (E) *Egr1* promoter-driven luciferase reporter assay in control and METTL3-knockdown HL-1 cells under OGD/R conditions. Cells were co-transfected with pGL3-EGR1-luc and pRL-TK plasmids for 36 h, and luciferase activity was measured as the firefly/Renilla ratio (n = 4). (F) RT-qPCR analysis of *Egr1* precursor mRNA levels in control and METTL3-knockdown HL-1 cells under normoxic or OGD/R conditions (n = 3). (G, H) HL-1 cells were treated with actinomycin D (5 μg/mL) to inhibit transcription. *Egr1* precursor (G) and mature (H) mRNA levels were analyzed in control and METTL3-knockdown cells at the indicated time points (n = 3). (I, J) HL-1 cells were treated with cycloheximide (100 μg/mL) for the indicated time periods. Western blotting (left) and densitometric quantification (right) of EGR1 protein in HL-1 cells were performed to assess EGR1 protein stability (n = 3). (K) Representative polysome profiling of control and METTL3-knockdown HL-1 cells under OGD/R conditions. (L) Distribution of *Egr1* mRNA across non-ribosomal (<40S), 40S, 60S, 80S, and polysome fractions in control and METTL3-knockdown HL-1 cells under OGD/R conditions (n = 3). (M, N) Representative immunofluorescence images of EGR1 protein expression in control and METTL3-knockdown HL-1 cells under OGD/R conditions (n = 3). Scale bar: 100 μm. All data are presented as mean ± SD. ns indicates not significant; **P* < 0.05; ***P* < 0.01; ****P* < 0.001; *****P* < 0.0001.

**Supplementary Table I. Antibodies used for WB.**

| Antibodies | Concentration | Source | Manufacturer | Cat. number |
| --- | --- | --- | --- | --- |
| METTL3 | 1:1000 | Rabbit | Abcam | ab195352 |
| YTHDF1 | 1:1000 | Rabbit | Abcam | ab220162 |
| IGF2BP2 | 1:1000 | Rabbit | Proteintech | 11601-1-AP |
| c-caspase-3 | 1:2000 | Rabbit | Cell Signaling Technology | 9664S |
| Caspase-3 | 1:2000 | Rabbit | Cell Signaling Technology | 9662S |
| Caspase-8 | 1:1000 | Rabbit | Proteintech | 13423-1-AP |
| RIPK1 | 1:1000 | Rabbit | Affinity | AF7877 |
| p-RIPK1 (Ser166) | 1:1000 | Rabbit | Affinity | AF2398 |
| MLKL | 1:1000 | Rabbit | Proteintech | 21066-1-AP |
| p-MLKL (Ser358) | 1:1000 | Rabbit | Affinity | AF7420 |
| GSDMD | 1:1000 | Rabbit | Affinity | AF4012 |
| RIPK3 | 1:1000 | Rabbit | Cell Signaling Technology | #10188T |
| Caspase-1 | 1:1000 | Rabbit | Abmart | T510200 |
| ZBP1 | 1:1000 | Rabbit | Cell Signaling Technology | 33402T |
| EGR1 | 1:1000 | Rabbit | Cell Signaling Technology | 4154T |
| ATF3 | 1:1000 | Rabbit | ABclonal | A13469 |
| LDHA | 1:1000 | Rabbit | ABclonal | A1146 |
| p300 | 1:500 | Rabbit | Affinity | AF5360 |
| eEF1A1 | 1:1000 | Rabbit | ABclonal | A23515 |
| eEF2 | 1:1000 | Rabbit | ABclonal | A9721 |
| L-Lactyl Lysine | 1:1000 | Rabbit | PTM BIO | PTM-1401 |
| L-Lactyl-Histone H3 (Lys18) | 1:1000 | Rabbit | PTM BIO | PTM-1406RM |
| Histone H3 | 1:5000 | Rabbit | PTM BIO | PTM-1001RM |
| β-actin | 1:10000 | Mouse | Proteintech | HRP-60008 |
| GAPDH | 1:10000 | Mouse | Proteintech | 60004-1-Ig |
| Mouse Anti-rabbit IgG | 1:2000 | Mouse | Cell Signaling Technology | #5127 |
| Goat Anti-Rabbit IgG H&L | 1:10000 | Goat | Abcam | ab6721 |

**Supplementary Table II. Primers for RT-qPCR.**

| Gene | Primer Sequence |
| --- | --- |
| *Mettl3(Mouse)* | 5’-CGCTGCCTCCGATGTTGATCTG-3’  5’-TCTCCTGACTGACCTTCTTGCTCTG-3’ |
| *METTL3(Human)* | 5’-CTGTGTCCATCTGTCTTGCCATCTC-3’  5’-ACCTCGCTTTACCTCAATCAACTCC-3’ |
| *Aim2(Mouse)* | 5’-GTCACCAGTTCCTCAGTTGTG-3’  5’-CACCTCCATTGTCCCTGTTTTAT-3’ |
| Mefv(Mouse) | 5’-TCATCTGCTAAACACCCTGGA-3’  5’-GGGATCTTAGAGTGGCCCTTC-3’ |
| Nlrp3(Mouse) | 5’-ATTACCCGCCCGAGAAAGG-3’  5’-TCGCAGCAAAGATCCACACAG-3’ |
| Nlrc4(Mouse) | 5’-TGCTGAAGCTACACAAGGAGGTC-3’  5’-TCCGTGGTGGTGGTGACAATG-3’ |
| Zbp1(Mouse) | 5’-AAGAGTCCCCTGCGATTATTTG-3’  5’-TCTGGATGGCGTTTGAATTGG-3’ |
| Tlr3(Mouse) | 5’-GTGAGATACAACGTAGCTGACTG-3’  5’-TCCTGCATCCAAGATAGCAAGT-3’ |
| Mda5(Mouse) | 5’-AGATCAACACCTGTGGTAACACC-3’  5’-CTCTAGGGCCTCCACGAACA-3’ |
| Nlrp6(Mouse) | 5’-CTCGCTTGCTAGTGACTACAC-3’  5’-AGTGCAAACAGCGTCTCGTT-3’ |
| Nlrp12(Mouse) | 5’-GCCCTCACGGTCCACTTTCTG-3’  5’-TCCATGCCAACACTTCCTCCTTC-3’ |
| Mettl14(Mouse) | 5’-CTGAGAGTGCGGATAGCATTG-3’  5’-GAGCAGATGTATCATAGGAAGCC-3’ |
| Wtap(Mouse) | 5’-GAACCTCTTCCTAAAAAGGTCCG-3’  5’-TTAACTCATCCCGTGCCATAAC-3’ |
| Virma(Mouse) | 5’-TCCAGCGGTGTCCAGATGTTAG-3’  5’-AGAGGTTCAAGCCATTTGCCAAG-3’ |
| Rbm15(Mouse) | 5’-CGAGTCCGCTGTGTGAAAC-3’  5’-TCCCCACGAGAACTGGAGTC-3’ |
| Alkbh5(Mouse) | 5’-CGCGGTCATCAACGACTACC-3’  5’-ATGGGCTTGAACTGGAACTTG-3’ |
| Fto(Mouse) | 5’-TTCATGCTGGATGACCTCAATG-3’  5’-GCCAACTGACAGCGTTCTAAG-3’ |
| Atf3(Mouse) | 5’-GAGGATTTTGCTAACCTGACACC-3’  5’-TTGACGGTAACTGACTCCAGC-3’ |
| ATF3(Human) | 5’-GAGGATTTTGCTAACCTGAC-3’  5’-CTACCTCGGCTTTTGTGATGG-3’ |
| Egr1(Mouse) | 5’-CCTTTTCTGACATCGCTCTGAA-3’  5’-CGAGTCGTTTGGCTGGGATA-3’ |
| EGR1(Human) | 5’-ACCTGACCGCAGAGTCTTTTC-3’  5’-GATGAGCTGGGACTGGTAGC-3’ |
| Dusp1(Mouse) | 5’-GTTGTTGGATTGTCGCTCCTT-3’  5’-TTGGGCACGATATGCTCCAG-3’ |
| DUSP1(Human) | 5’-AGTACCCCACTCTACGATCAGG-3’  5’-GAAGCGTGATACGCACTGC-3’ |
| Hspa1a(Mouse) | 5’-TGGTGCAGTCCGACATGAAG-3’  5’-GCTGAGAGTCGTTGAAGTAGGC-3’ |
| HSPA1A(Human) | 5’-AGCTGGAGCAGGTGTGTAAC-3’  5’-CAGCAATCTTGGAAAGGCCC-3’ |
| Gadd45a(Mouse) | 5’-CCGAAAGGATGGACACGGTG-3’  5’-TTATCGGGGTCTACGTTGAGC-3’ |
| GADD45A(Human) | 5’-CGAAAGGATGGATAAGGTG-3’  GGATCAGGGTGAAGTGGA-3’ |
| Nr4a1(Mouse) | 5’-GTGCAGTCTGTGGTGACAATG-3’  5’-TCCGCCGCCTCTTGTCCAC-3’ |
| NR4A1(Human) | 5’-TGCTGTGTGTGGGGACAACG-3’  5’-TTTCGCCGCCTCTTGTCCAC-3’ |
| Ep300(Mouse) | 5’-TTCAGCCAAGCGGCCTAAA-3’  5’-CGCCACCATTGGTTAGTCCC-3’ |
| Mtor(Mouse) | 5’-ACCGGCACACATTTGAAGAAG-3’  5’-CTCGTTGAGGATCAGCAAGG-3’ |
| MTOR(Human) | 5’-CATTCCGACCTTCTGCCTTCAC-3’  5’-CGTTCCTTCTCCTTCTTGACACAG-3’ |
| Met(Mouse) | 5’-GTGAACATGAAGTATCAGCTCCC-3’  5’-TGTAGTTTGTGGCTCCGAGAT-3’ |
| MET(Human) | 5’-GCCACGGGACAACACAATACAG-3’  5’-AAGTGCCACCAGCCATAGGAC-3’ |
| Prkaa1(Mouse) | 5’-GTCAAAGCCGACCCAATGATA-3’  5’-CGTACACGCAAATAATAGGGGTT-3’ |
| PRKAA1(Human) | 5’-TGCCAAAGGAGTGATTCAGATGC-3’  5’-GGTCAACAGGAGAAGAGTCAAGTG-3’ |
| Cd274(Mouse) | 5’-GCTCCAAAGGACTTGTACGTG-3’  5’-TGATCTGAAGGGCAGCATTTC-3’ |
| CD274(Human) | 5’-GTAAGACCACCACCACCAATTCC-3’  5’-TTAGTTGTTGTGTTGATTCTCAGTGTG-3’ |
| Pre-Egr1(Mouse) | 5’-ACATCGGGAGTCAGTGGTAG-3’  5’-GTTACTGGAGAGGAATCACTGG-3’ |
| Ythdf1(Mouse) | 5’-ACAGTTACCCCTCGATGAGTG-3’  5’-GGTAGTGAGATACGGGATGGGA-3’ |
| Igf2bp1(Mouse) | 5’-CGGCAACCTCAACGAGAGT-3’  5’-GTAGCCGGATTTGACCAAGAA-3’ |
| Igf2bp2(Mouse) | 5’-GTCCTACTCAAGTCCGGCTAC-3’  5’-CATATTCAGCCAACAGCCCAT-3’ |
| Igf2bp3(Mouse) | 5’-CCTGGTGAAGACGGGCTAC-3’  5’-TCAACTTCCATCGGTTTCCCA-3’ |
| Elavl1(Mouse) | 5’-GGATGACATTGGGAGAACGAAT-3’  5’-TGTCCTGCTACTTTATCCCGAA-3’ |
| Fluc | 5’-CGAGGCTAAGGTGGTGGACTTGG-3’  5’-CCAGCCGTCCTTGTCGATGAGAG-3’ |
| Rluc | 5’-TGTCCGCAACTACAACGCCTACC-3’  5’-GCGTCCTCCTGGCTGAAGTGG-3’ |

**Supplementary Table III. Primers for ChIP-qPCR.**

| Mettl3(Mouse) | 5’-TACACCCACCCGTCAAACAC-3’  5’-GCCGGATAGAGCCAAGATGA-3’ |
| --- | --- |
| Zbp1(Mouse) | 5’-AGTGTCTGAGCTGTTCACGG-3’  5’-AGCCCTGTGAGGAGGAAGAT-3’ |

**Supplementary Table IV. Antibodies used for immunofluorescent staining.**

| Antibodies | Concentration | Source | Manufacturer | Cat. number |
| --- | --- | --- | --- | --- |
| METTL3 | 1:200 | Rabbit | Abcam | ab195352 |
| EGR1 | 1:200 | Rabbit | Cell Signaling Technology | 4154T |
| L-Lactyl-Histone H3 (Lys18) | 1:200 | Rabbit | PTM BIO | PTM-1406RM |
| YSFluor™ 488 Goat Anti-Rabbit IgG (H+L) | 1:2000 | Goat | YEASEN | 33106ES60 |
| YSFluor™594 Rabbit Anti-Mouse IgG (H+L) | 1:2000 | Rabbit | YEASEN | 33912ES60 |

**Supplementary Table V. siRNAs used for cell tranfection.**

| Gene | siRNA Sequence |
| --- | --- |
| *Negative control* | 5′-UUCUCCGAACGUGUCACGUTT-3′ |
| *Mettl3(Mouse)* | 5′-GGUUCGUUCCACCAGUCAUTT-3′ |
| *METTL3(Human)* | 5′-GCAAGAAUUCUGUGACUAUTT-3′ |
| *Ythdf1(Mouse)* | 5′-GCCCACAGCUAUAACCCUAAATT-3′ |
| *Igf2bp2(Mouse)* | 5′-GGGUAAAGUGGAAUUGCAUTT-3′ |
| *Zbp1(Mouse)* | 5′-CAGCCUAGCCUUGAUGAAATT-3′ |
| *Egr1(Mouse)* | 5′-CACUCCACUAUCCACUAUUAATT-3′ |
| *EGR1(Human)* | 5′-CCAUGGACAACUACCCUAATT-3′ |
| *Ep300(Mouse)* | 5′-CAAUUCCGAGACAUCUUGAGATT-3′ |
| *Ldha(Mouse)* | 5′-CGUGAACAUCUUCAAGUUCAUTT-3′ |
